# The influence of the practice environment on sharing decision making between older persons and nurses in residential aged care: a qualitative person-centred study

**DOI:** 10.3389/frhs.2026.1769782

**Published:** 2026-06-18

**Authors:** Kelly Marriott-Statham, Michele Hardiman, Alison Wood, Caroline Dickson

**Affiliations:** 1 Centre for Person-centred Practice Research, Queen Margaret University, Edinburgh, United Kingdom; 2 Centre for Ageing, Research and Translation, University of Canberra, Bruce, ACT, Australia; 3 Blackrock Health, Galway Clinic, Galway, Ireland

**Keywords:** nursing, older persons, person-centred practice, practice environment, residential aged care, sharing decision making

## Abstract

**Introduction:**

With an ageing population and global attention on how older people are cared for, there is a need to understand how older persons are enabled to participate in care decisions. Enabling and sharing decision making is promoted widely in healthcare, yet evidence demonstrates that shared decision making processes are not effectively translated into practice. The healthcare practice environment plays a key role in shaping how older people are included in their care. Further, residential aged care environments can be more complex, as paternalistic attitudes, rigidity and routine, resourcing, and workplace culture impact person-centred practice and sharing decision making processes. The aim of this research was to explore how the practice environment influences sharing decision making between older persons and nurses in residential aged care.

**Methodology and methods:**

Using person-centred methodology, older persons and nurses were recruited from an Australian residential aged care setting. Information was generated through emotional touchpoint interviews (*n* = 17; older persons *n* = 5; nurses *n* = 6), observations of practice (*n* = 8), and practice development methods. Creative hermeneutic analysis was used to iteratively interpret the information generated with participants to develop and refine themes, and reflexive journalling and participant dialogue supported analytic rigour.

**Findings:**

Seven components were found to influence the relational connectedness and sharing decision making between older persons and nurses: affirming personhood, reciprocal trustworthiness, time as presence, intentional way of being, negotiating relational boundaries, organisational values in action, and policies in practice. This research contributes a new perspective of sharing decision making as a relational process continuously influenced by the people, processes, and structures within the practice environment.

**Conclusions and implications:**

The practice environment was identified as key to how decision making occurs between older persons and nurses. To support sharing decision making, aged care organisations need flexible policies and workforce models that prioritise relationship building. Nurses should be supported to develop reflective and power sharing practices to enhance emotional intelligence, and have the time to connect meaningfully with older persons. Creating conditions where older people are respected, heard, and genuinely involved in care must be embedded within the elements of the practice environment of residential aged care.

## 1 Introduction

Choice and decision making are central to human rights-based and person-centred care of older persons living in residential aged care. Around the world, upholding autonomy and respecting older persons' preferences are deeply embedded in quality standards, polices, and legislation (1–4). Despite this recognition, older persons living in residential aged care continue to experience limited autonomy and participation in decisions about their everyday care. In Australia, the Royal Commission into Safety and Quality in Aged Care similarly identified systemic issues that disempower older persons and recommended significant reforms to embed high-quality person-centred care in aged care services (5). Person-centred care is a well-recognised concept informing global health care strategy and policy. The focus of person-centred care is humanising healthcare by placing the person at the centre of care delivery and experience, with recognition of their uniqueness as a person with preferences, values and beliefs (3, 6, 7). Shared decision making is core to person-centred care, which is one way these principles can be translated into everyday practice.

Shared decision making is a concept that has gained global attention over the past decade. The collaborative approach aims to enhance autonomy and engagement by involving persons in decisions about their care, rather than practitioners making decisions on their behalf (1, 8, 9). Within healthcare literature, the terms *shared* and *sharing* decision making are both used. *Shared* decision making has origins in patient-centred care and is recognised as a dyadic interaction between doctor and patient, usually as a one-off treatment decision (9–11). By contrast, person-centred care extends beyond the treatment decision to include the whole person, their values, beliefs, preferences, hopes for the future, and other people significant to them (7, 12, 13). *Sharing* decision making reflects person-centredness by acknowledging decision making as an ongoing, relational process that promotes autonomy and the fundamental right to self-determination (8, 14). Sharing decision making permeates and enhances all aspects of person-centred practice (8, 15, 16).

Nurses are increasingly more engaged in, and initiating, sharing decision making with persons in care, and are perceived as valuable and integral to decision making processes (14, 17–21). In residential aged care, sharing decision making is especially important as autonomy for older persons is upheld through smaller everyday decisions, to bigger, rarer decisions that influence wellbeing and quality of life (22). Within Australian residential aged care, Registered Nurses and Care Support Workers are the constant point of nursing care for older persons. Registered Nurses are regulated healthcare professionals who lead and coordinate care, conduct assessments, develop care plans, and provide evidence-informed care with older persons (23–27). Care Support Workers (also known as Personal Care Workers or Assistants in Nursing) provide physical, emotional, and social support, including assistance with daily activities, such as mobility and personal care (28, 29). Although Care Support Workers are not a regulated profession, they work closely with Registered Nurses, and both have an important role in enabling autonomy through everyday decision making with older persons (27, 29, 30).

Despite the importance of sharing decision making, evidence demonstrates it is not consistently or effectively translated into practice (9, 11, 21, 31, 32). Residential aged care adds further complexity because decision making occurs within environments shaped by routines, resourcing constraints, organisational cultures, and regulatory requirements (1, 33–35). These conditions can create or reinforce power imbalances between older persons and healthcare practitioners. Older persons' autonomy can be limited by ageist assumptions, institutional routines, professional hierarchies, and risk adverse cultures that foregrounds dependency and need, instead of capability and choice (22, 34, 36–38). Nurses may also experience constrained professional autonomy within hierarchical and highly regulated care environments, with limited opportunities to engage in decision making processes due to workload, while simultaneously perceived as holding more power in interactions with older persons (1, 36, 39, 40). Understanding sharing decision making, therefore, requires attention to the practice environment and what enables or constrains autonomy.

Little is known about how sharing decision making occurs between older persons and nurses, or how residential aged care environments influence the process (21). The specific aspects of the practice environment that shape sharing decision making is underexplored, and the older person's voice is largely absent from the literature (21). The aim of this research was to explore how the practice environment influences sharing decision making between older persons and nurses in residential aged care. The research questions were: *How do nurses enable older persons to participate in sharing decision making?* and; *How does the practice environment influence an older person's ability to participate in sharing decision making?* To address these questions, the study was informed by a theoretical perspective that positions sharing decision making as relational and contextually influenced.

## 2 Theoretical positioning

This research is underpinned by a theoretical perspective that guided its design (14). The key concepts forming this perspective were drawn from the research aim and include sharing decision making, the practice environment and older persons and nurses in residential aged care.

Firstly, sharing decision making was understood through the lens of person-centred literature, where its origins are from the philosophical contributions of Gadow (41), Gilligan (42), and McCormack (43, 44). Sharing decision making is defined as “the facilitation of involvement about decision making by patients and others significant to them by considering values, experiences, concerns, and future aspirations” (22, p. 54), which is the perspective adopted for this research. The term *sharing*, rather than *shared*, reflects decision making as an ongoing and relational process.

**Figure 1:**
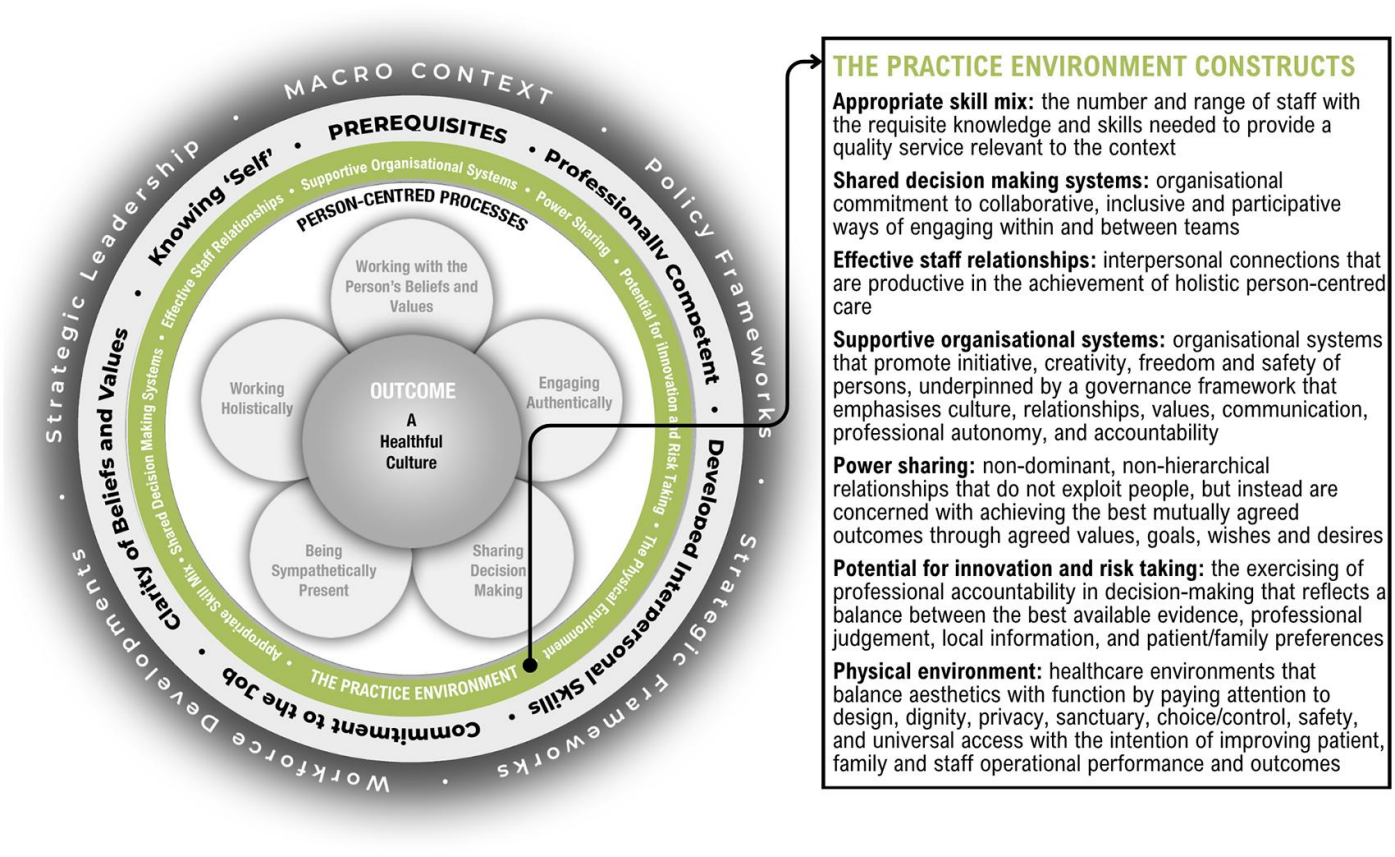
The practice environment domain constructs within the Person-centred Practice Framework, including definitions [image colouring adapted from McCance et al. (47); image licensed under CC by 4.0; definitions reproduced with permission from McCance and McCormack (48)].

The second concept is the practice environment, where sharing decision making takes place. The term is drawn from the Person-centred Practice Framework, a middle-range theory created as a way of viewing and operationalising person-centred practice in healthcare (45–47). The practice environment refers to the “people, processes and structures” (43, p. 27) of the context and is identified as having the greatest influence on person-centred practice, including sharing decision making (15, 16, 35, 48, 49). Seven constructs make up the practice environment domain (Figure 1) (*appropriate skill mix; shared decision making systems; effective staff relationships; supportive organisational systems; power sharing; potential for innovation and risk taking;* and *the physical environment*), and are derived from earlier empirical work by Rycroft-Malone et al. (50) and McCormack et al. (51). The practice environment domain reflects the multifaceted and complex nature of health care contexts worldwide, which is an important perspective for this research.

Finally, older persons and nurses are positioned as relational beings, each bringing their own subjectivity and experiences to their interactions. Philosopher, Martha Nussbaum, has extensive work on emotions and capabilities supporting human dignity (flourishing) that reinforce the understanding of persons as subjective, relational, and contextually placed (52–56). Additionally, both older persons and nurses are understood to be shaped by societal narratives of ageing and care, including ageism (37, 39, 56, 57) and hierarchical healthcare structures (36, 40, 58–60). These narratives contribute to the construction of knowledge and power by older persons and nurses, which are realised in residential aged care (context). These constructions of knowledge and power create cycles of oppression that perpetuate power imbalances and feelings of disempowerment for both older persons and nurses, limiting autonomy, reducing care quality, and undermining wellbeing (36, 39, 40, 59, 60). Brian Fay's critical social science reflects the view that knowledge and power are socially constructed and enacted (61, 62). Together, these ideas frame older persons and nurses as relational actors within a complex social and political context (residential aged care) that shapes how decision making is engaged with, and how care is experienced.

The theoretical perspective views sharing decision making as a relational, contextually influenced process, shaped by emotional, social, and political realities of older persons and nurses. This theoretical perspective informed the methodology, choice of methods and the interpretation of how the practice environment influences sharing decision making between older persons and nurses in residential aged care for this research.

## 3 Methodology and methods

This research was guided by a person-centred methodology and was informed by the theoretical perspective described in the previous section. This section describes the research design, study setting, participants and recruitment, and the specific methods used for information (data) generation, collection and analysis.

### 3.1 Research design

**Figure 2:**
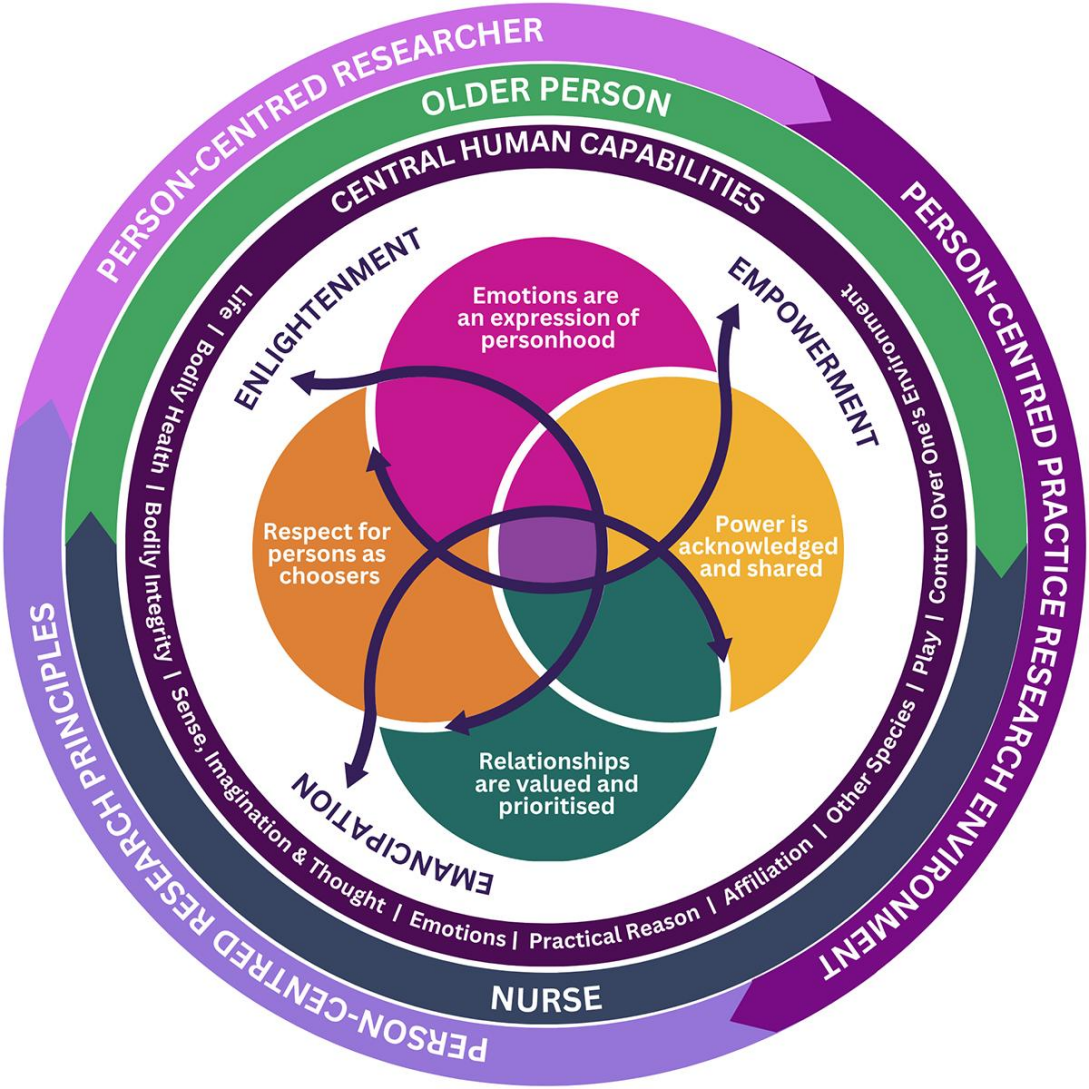
Methodological framework.

A person-centred methodology was designed for this research, informed by the theoretical perspective, research aim and the residential aged care context (14). The methodology encompassed person-centred research principles (63), Nussbaum's Central Human Capabilities (52), and Fay's concepts of enlightenment, empowerment, and emancipation (61), which forms the methodological framework for this research presented in Figure 2.

The person-centred research principles: connectivity, attentiveness and dialogue, empowerment and participation, and critical reflexivity; formed the methodological foundation for relational and contextual engagement throughout the research (63). These principles guided recruitment, ongoing consent processes, relationship building, and the use of participatory methods to ensure participants were involved in ways that supported authenticity and active participation. Nussbaum's capabilities framework offered a way of recognising persons subjectively (rather than objectively) in a context where autonomy can be constrained (52, 54). Capabilities such as affiliation and practical reason enabled attention to dignity, agency, choice, and participation during information generation and analysis (52, 54, 55). Fay's ideas on enlightenment, empowerment, and emancipation formed the final methodological foundation to recognise relational and structural power imbalances in residential aged care (5, 61). These ideas informed the choice of information generation methods and analysis to support attention to organisational norms and constraints to challenge oppressive constructs within the practice environment (61, 64). These three foundations created an ethical and relationally grounded methodology and informed the choice of methods for this research.

**Figure 3:**
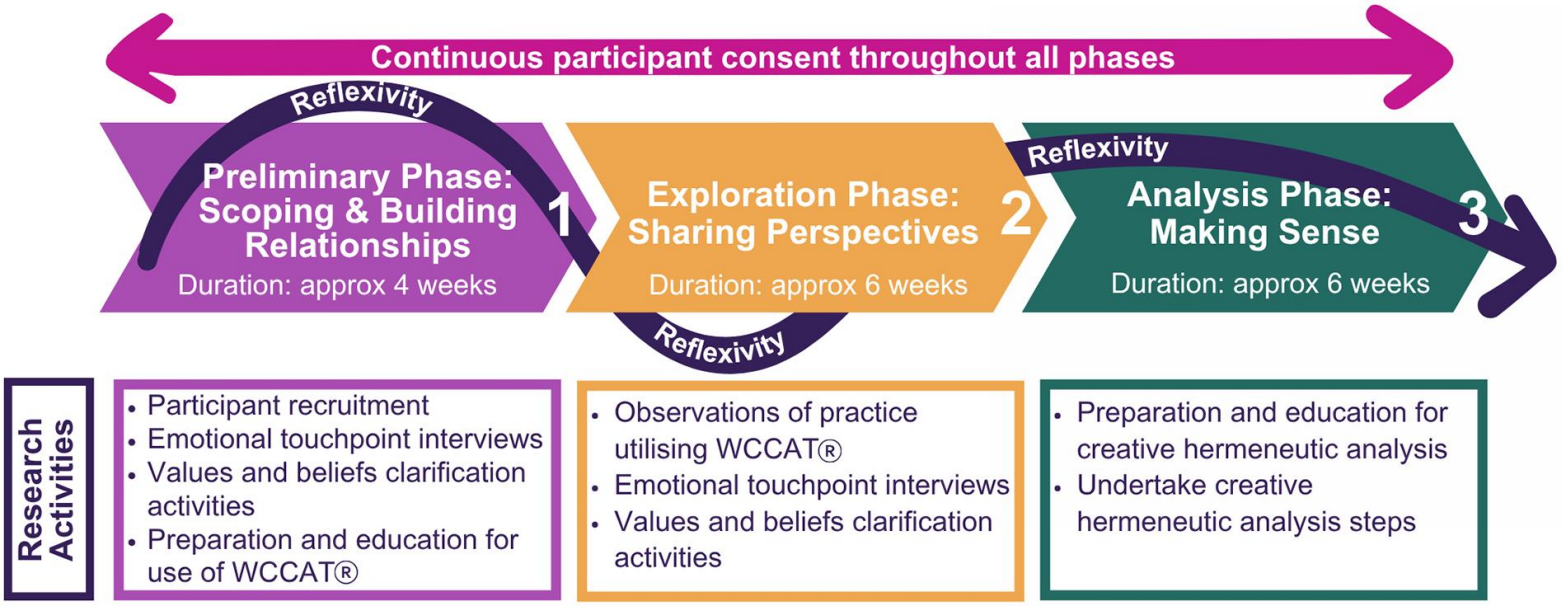
Research phases and activities (WCCAT® = Workplace Culture Critical Analysis Tool).

Research activities were organised into three connected phases (Figure 3) to maximise participation and embed reflexivity. Firstly, the *Preliminary Phase* focused on building trust and relationships within the research context, the *Exploration Phase* involved generating information from multiple perspectives, and the final *Analysis Phase* centred on synthesising information with participants. This study was undertaken as part of the first author's doctoral research at Queen Margaret University, Edinburgh, United Kingdom (14). Ethical approval was granted by the Queen Margaret University Research Ethics Panel, with reciprocal approval obtained from the University of Wollongong Human Research Ethics Committee in Australia (Approval Number 2022/373).

### 3.2 Study setting, participants, and recruitment

This research was conducted in a residential aged care setting in regional Australia, operated by a large Christian aged care services provider. At the time this study took place (February to July 2022), mandated face mask wearing was still in effect in residential aged care settings due to the COVID-19 pandemic. Older persons living in the setting and nurses employed there were eligible to participate. A minimum length of residence for older persons or duration of employment for nurses was not specified as the study focused on experiences of sharing decision making among persons living or working in the setting. Detailed inclusion and exclusion criteria for both participant groups are summarised in Table 1. Family members, organisational leaders, and other healthcare practitioners were not recruited as participants, as the study focused on sharing decision making between older persons and nurses. However, their influence was considered where it appeared in participant experiences.

**Table 1:** Participant inclusion and exclusion criteria.

Participant group	Inclusion criteria	Exclusion criteria
Older persons	Speaks English	Does not speak English
	Lives permanently or on respite within the residential aged care setting	Unable to provide written, verbal, or process consent and/or has a substitute decision-maker appointed
	Able to provide written, verbal, or process consent	Aged <65 years, or <50 years if identifying as an Aboriginal or Torres Strait Islander person^a^
	Aged ≥65 years, or ≥50 years if identifying as an Aboriginal or Torres Strait Islander person^a^	
Nurses	Employed in a permanent, part-time, or casual role within the residential aged care setting	Working at the residential aged care setting through an external agency
	Employed as a Registered Nurse, Enrolled Nurse, or Care Support Worker whose role involves direct relational contact and support of older persons	Employed in a role other than Registered Nurse, Enrolled Nurse, or Care Support Worker
		Care Support Workers whose role does not involve direct personal care (e.g., not providing support with hygiene, meals, mobility, medications, or other personal care tasks)

a The age criterion for older persons is consistent with Australian aged care eligibility criteria, where Aboriginal and Torres Strait Islander peoples are eligible from 50 years of age in recognition of lower life expectancy and earlier age-related health and care needs.

Recruitment occurred primarily in the *Preliminary Phase* of this research (Figure 3) and followed ethical and person-centred principles to minimise coercion and support informed choice. Participation was voluntary, with assurances that involvement would not influence care or employment, and participants could withdraw at any time without explanation. Consistent with the methodological design, recruitment was guided by participants' willingness to contribute across the phases; instead of saturation or statistical generalisability being achieved. Study information was provided through posters in common areas and in routine resident and staff meetings, where the study was introduced and participant information sheets were available. Potential participants were able to ask questions in person, by phone, or over email directly to the research team, or to an independent advisor not directly involved in the research. Written consent was obtained before participation and process consent was used throughout to ensure ongoing assent, recognising consent as an ongoing process (65–67). The recruitment approach upheld autonomy, choice and relational decision making, consistent with the person-centred methodology. For example, participants could consult family before agreeing, choose involvement in one or multiple aspects of the research, and were asked verbally for consent during participation. Observers also monitored for signs of discomfort to ensure participation remained voluntary.

### 3.3 Information (data) collection

Information (data) generation and collection occurred across the *Preliminary* and *Exploration* phases of the study (Figure 3), from February to June 2022. The term *information* is preferred over *data*, as it better reflects a person-centred approach to this research. *Information* encompasses the rich, contextual, and subjective insights from the research setting, whereas *data* implies a detached, factual perspective often associated with quantitative methods (14). Multiple methods of information generation and collection were chosen, informed by the relational and participatory principles of the methodology for this research. The methods included values and beliefs clarification activities, emotional touchpoint interviews, and observations of practice. Reflexive, creative journalling was also undertaken throughout the research to document the first author's developing interpretations. The methods created multiple perspectives and insights into sharing decision making processes within the residential aged care setting. Each method and its application to this research is explained in the following section.

#### 3.3.1 Values and beliefs clarification activities

Values and beliefs clarification activities are practice development tools designed to explore assumptions, values, and beliefs in practice (68). This method creates opportunities for reflection and discussion on the purpose of a practice, the factors that enable or hinder it, and related beliefs (68). The activity raises awareness of shared values and beliefs among participants and supports understanding of the workplace culture within a practice environment (68, 69).

In this study, values and beliefs clarification activities were conducted during the *Preliminary* and *Exploration* phases to explore understandings of person-centred care and sharing decision making. Large sheets with open-ended statements [e.g., *The purpose of (person-centred care/sharing decision making) is…; This purpose can be achieved by… The factors that enable/hinder this purpose are…*] were placed in a common area for 2 weeks, and older persons and nurses were invited to contribute responses to each of the statements in their own time. This inclusive approach minimised disruption to routines (68, 70). Responses were then themed with a group of nurses and shared back to both participant groups to explore how values and beliefs were enacted and experienced in practice.

#### 3.3.2 Emotional touchpoint interviews

Emotional touchpoint interviewing is a technique that enables a person to identify and share emotions linked to a specific healthcare experience (71). Participants are asked to reflect on a particular touchpoint (experience) and select from positive and negative emotion words displayed on cards. Blank cards are provided for emotions not provided. This approach assists persons to articulate feelings beyond words such as *“good”* or *“fine”* by offering a range of response options (72, 73). Emotional touchpoint interviewing provides a structured way for persons to reflect on and communicate their emotions, offering valuable insights into their experiences.

Emotional touchpoint interviews were conducted with 11 participants (older persons *n* = 5; nurses *n* = 6), that generated 17 interviews across the *Preliminary* and *Exploratory* phases of the research (14). Interviews were conducted in person and explored perceptions of living or working in the setting and experiences of participating in sharing decision making. Interviews were audio-recorded with consent, transcribed, de-identified, and returned to participants for review. Participants were informed before consenting how pseudonyms and role descriptors would be used when reporting the research. Participants were invited to choose a pseudonym; however, some participants expressed a preference to use their real name. A variation to the ethical approval was obtained to uphold the person-centred methodology and honour participants' choice in how their contributions were represented. Role descriptors were limited to broad participant categories, such as older person, Registered Nurse or Care Support Worker, to support interpretation of role-based perspectives while minimising identifiability.

#### 3.3.3 Observations of practice

Observation is a valuable method for understanding the culture of a workplace, including routines, interactions, and the practice environment (69, 74). The Workplace Culture Critical Analysis Tool (WCCAT®) is a systematic observational tool grounded within the Person-centred Practice Framework that enables the collection of information about patterns of practice and evidence of culture, informing initiatives for practice development (74, 75). The WCCAT® is a process of structured observation, feedback, and collaborative reflection that generates insights and co-creation of action plans to promote person-centred practice (74). This method offers an approach to generating and collecting information that aligns with the aim of this research by exploring what occurs in practice and how people experience the practice environment.

Observations of practice were undertaken in the *Exploration Phase* using the WCCAT® (*n* = 8) and focused on everyday care activities including handover, assistance with personal hygiene, mealtimes, medication rounds, and a leadership meeting (14). Before each observation, an explanation was provided to each potential participant about the process and purpose of the observation, and verbal consent was obtained. Observations were undertaken in pairs and lasted between 22 and 55 min, with notes captured on the WCCAT®. Following each observation, participants were invited to discuss and reflect with observers on patterns and insights noted by the observers, with discussions lasting between 20 and 70 min.

### 3.4 Information analysis

Creative hermeneutic analysis was chosen as the method for information analysis and was undertaken in the *Analysis Phase* of the research (Figure 3) from June to July 2022 (14). This approach enables meaning to surface at individual and group levels through subjective interpretation, ensuring that the findings are representative of the context and experiences of the persons the research is about (76–78). The flexible and iterative nature of this analytical method enabled adjustments to the depth and pace of engagement to accommodate the participants' needs and external factors, such as the COVID-19 outbreaks. Six participants (older persons *n* = 2; nurses *n* = 4) from the earlier phases voluntarily chose to continue their participation into the *Analysis Phase* as part of the ongoing consent process used throughout this study. Participant roles informed interpretation of the findings but were not used to develop separate role-based themes.

**Figure 4:**
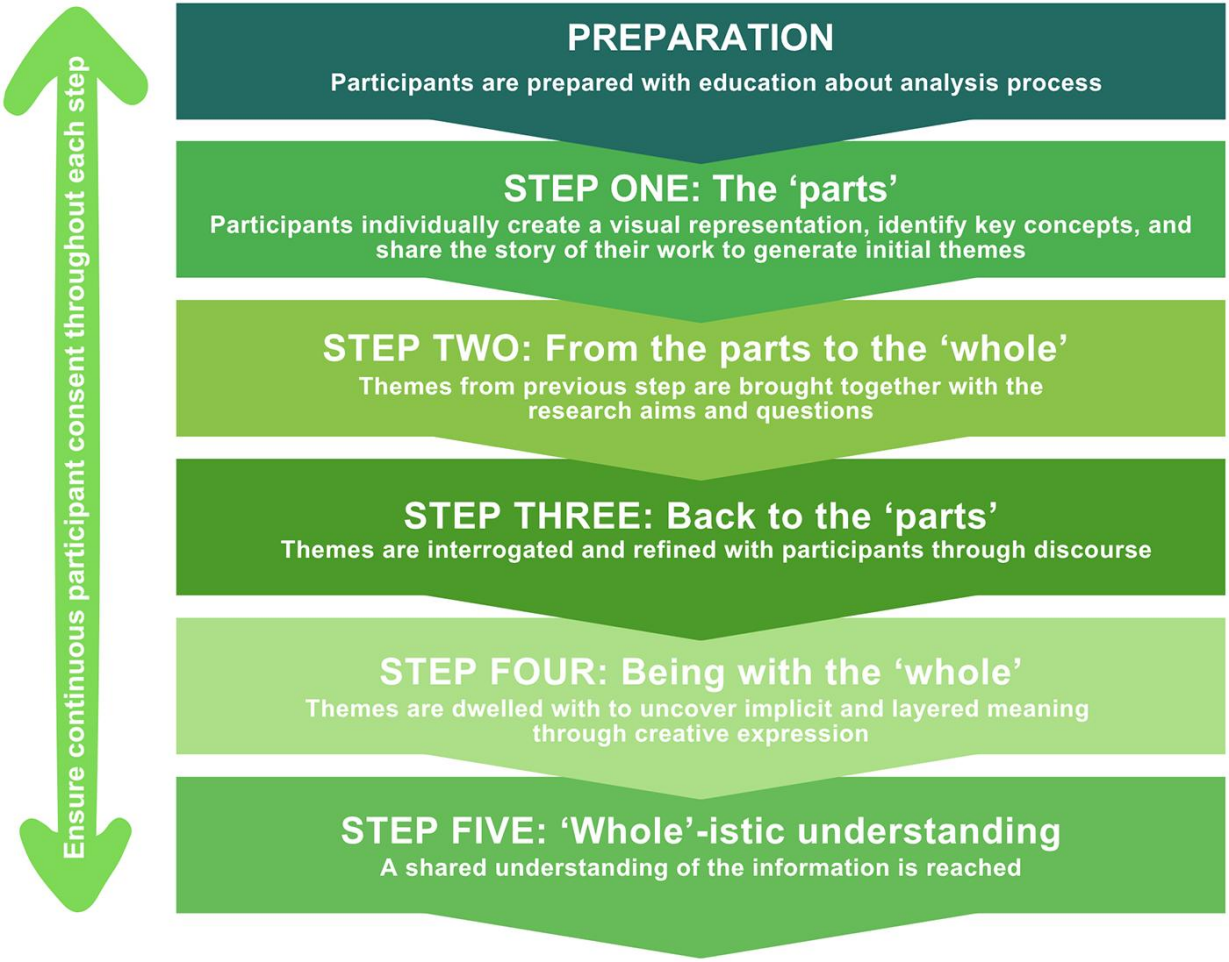
Creative hermeneutic circle analysis steps.

The steps for analysis in this research were adapted from Boomer and McCormack (77), Dickson et al. (76), and Sanders et al. (78) and are presented in Figure 4. The main adaptation was the inclusion of a *Preparation* step and specific references to the hermeneutic circle, acknowledging that understanding the “whole” is based on the interpretation of its individual “parts”, and vice versa (76). Each step supported participants to interpret meaning in their own personal context (horizon), and through dialogue, as a group (fusion of horizons) to develop a collective understanding of sharing decision making in residential aged care (79, 80). Journalling was undertaken by the first author to support analytic transparency and interpretations and was not included as an information source. Each step of the creative hermeneutic analysis and its application to this research is explained in the following section.

#### 3.4.1 Preparation

The purpose of this step was to prepare and orientate participants to the creative hermeneutic process and introduce key concepts, such as the hermeneutic circle and fusion of horizons. Each participant (*n* = 6) was prepared individually or in pairs, depending on their preferences and schedules. The research aim and questions were revisited, and the steps of creative hermeneutic analysis were explained using examples from other research (76–78). The relationships built in the earlier phases of the research promoted engagement with participants.

#### 3.4.2 Step one: the “parts”

**Figure 5:**
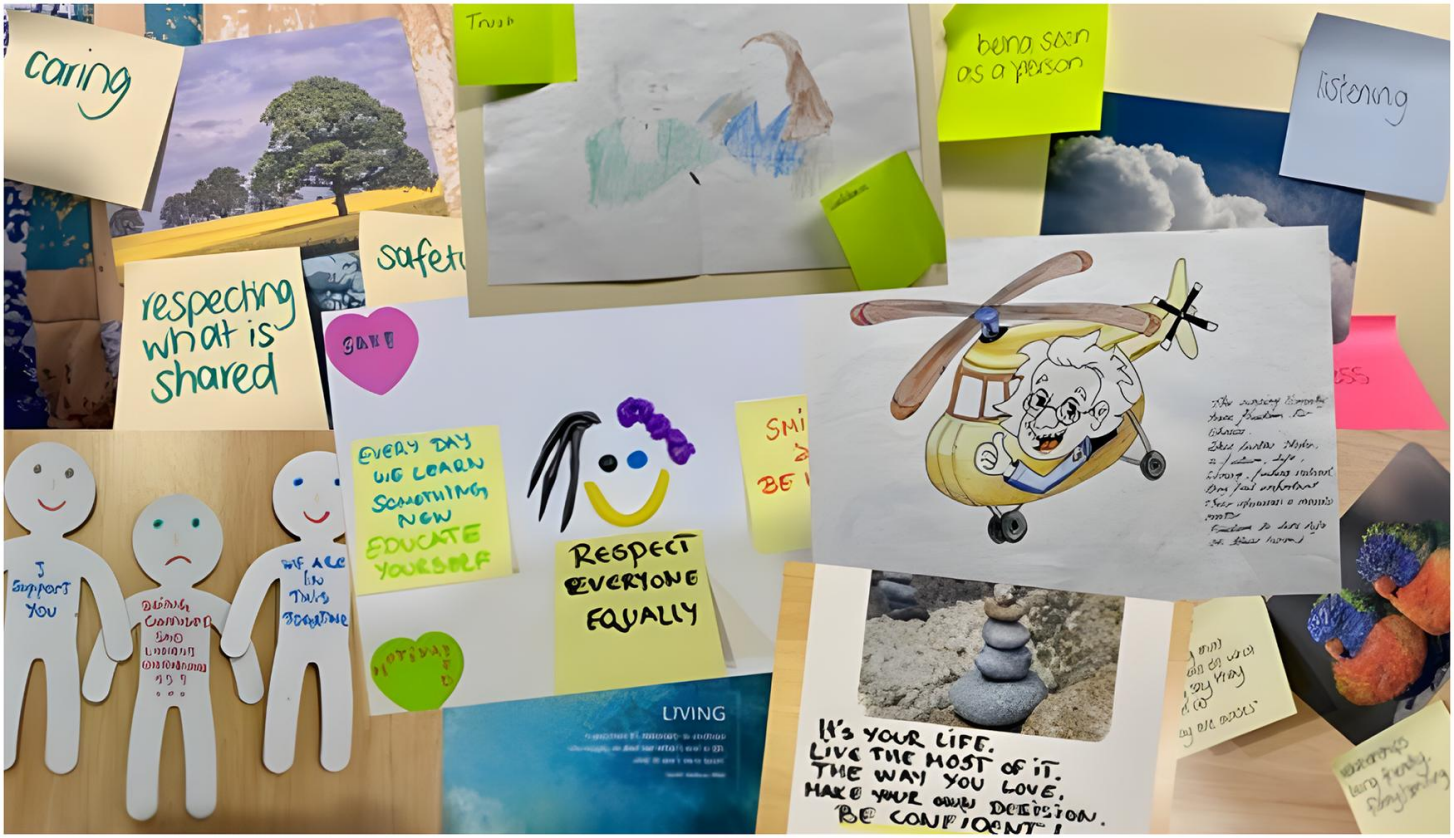
Collage of visual representations in step one of creative hermeneutic circle analysis.

In this first step of making sense of the information, individual interpretive insights were considered by focusing on the individual “parts” of the information (hermeneutic circle). Participants interpreted their own experiences to generate initial themes (individual horizons) by creating visual representations (collages, drawings, words) of their emotional touchpoint interviews and identified key ideas about what facilitated sharing decision making (Figure 5), then shared their story (individual horizons). For interview participants who did not participate in the creative hermeneutic analysis or preferred not to create visuals (*n* = 5), key ideas from their interviews were extracted and verified with them. These initial themes were treated as preliminary insights to be refined in the later steps of the analysis through movement between the “parts” and the “whole”.

#### 3.4.3 Step two: from the “parts” to the “whole”

Moving to the “whole” of the hermeneutic circle, the developing themes from the previous step were brought together with the WCCAT® observations, values and beliefs clarification activities, and the research aim and questions. Interview transcripts, WCCAT® observations and values and beliefs clarification activities were re-read together to compare, extend, challenge, and refine the developing themes. Supporting words, images, and excerpts were then added to each theme to show how meanings were constructed across information sources. This immersive “being with the whole” facilitated movement between individual horizons toward shared meaning.

#### 3.4.4 Step three: back to the “parts”

The “parts” were then revisited with participants to refine, interrogate, and verify the themes and their interpretations through dialogue. Participants reviewed the refined themes by reflecting on what resonated, what was missing, and how the themes related to living or working in the setting. Alternative interpretations were explored through dialogue, including whether themes overlapped, needed renaming, or should be combined. Dialogue enabled clarifying and collapsing of themes, for example, *safety* merged with the theme of *trust*. Themes became shared, representing a fusion of horizons.

#### 3.4.5 Step four: being with the “whole”

The research team then sat with participant-generated information sources, refined themes, reflexive journalling notes, philosophical and theoretical underpinnings, and creative representations to extend the depth of interpretation. Themes were retained when they resonated with the participants' interpretations, were evident across information sources, and helped explain how the practice environment shaped sharing decision making. Creative expression, including doodling, drawing, and writing, supported conceptualisation of how the developing themes related to one another, and a creative representation of the “whole” was developed.

#### 3.4.6 Step five: “whole”-istic understanding

A shared understanding about the meaning of the information was reached, named a “whole-istic understanding”, as a deliberate play on words referencing the “whole” of the hermeneutic circle. Verification with participants (*n* = 5; one older person had died) of the shared understanding of the whole was undertaken through dialogue and sharing refined themes, creative representation and the developing findings model. Through discourse, the placement and interrelationships of themes on the model was adjusted until consensus was reached about the “whole”-istic understanding of the influences of the practice environment on sharing decision making.

## 4 Findings

**Figure 6:**
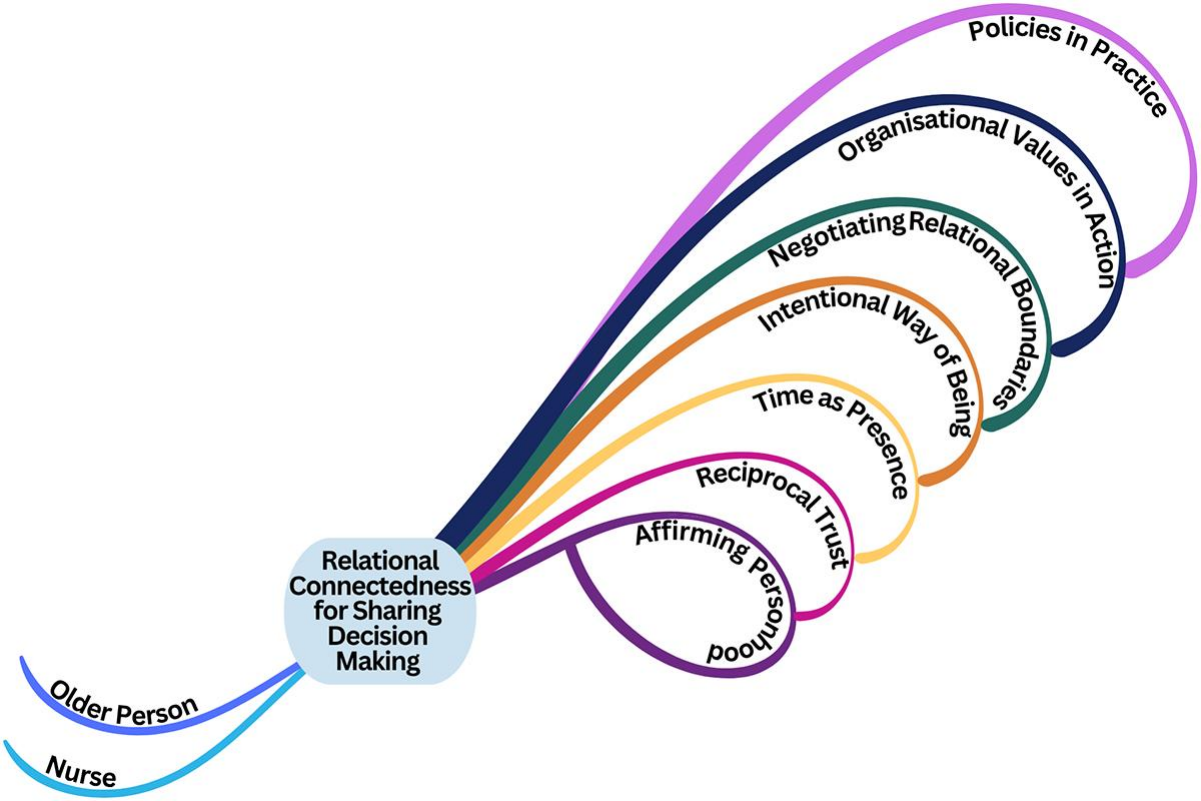
The threads influencing relational connectedness for sharing decision making between older persons and nurses.

The findings of this research are drawn from the multiple methods of information generation and collection, including: values and beliefs clarification activities [VBCA]; emotional touchpoint interviews [ETI], and; observations of practice using the Workplace Culture Critical Analysis Tool® [WCCAT]. The participants identified from all the information that *relational connectedness* was the foundation for which sharing decision making was possible in residential aged care. Older persons and nurses described being *in relation* as they both lived and worked in the residential aged care setting, but the degree to which the older person and nurse are connected is complex. Participants described relationships as a delicate balance, with many different and intertwining threads influencing how older persons and nurses engaged with one another when making decisions. Figure 6 is a visual representation of the influencing threads, with relational connectedness at the base and seven threads (*affirming personhood, reciprocal trust, time as presence, intentional way of being, negotiating relational boundaries, organisational values in action,* and *policies in practice*) intertwining to strengthen or weaken the relational connectedness between the older person and nurse. *Affirming personhood* is the anchoring thread that holds the others together.

Participants' visual representations created during analysis illustrated the relational nature of decision making between the older person and nurse. Joe (older person) selected an image of two birds sitting in parallel, looking in the same direction to depict the relationship and shared goals of the older person and nurse relationship (Figure 5). Similarly, Jess (Care Support Worker) sketched an older person and nurse side-by-side to portray a partnership, working together as equals (Figure 5). Observations of practice also demonstrated relational connectedness. During a medication round, a nurse gently touched an older person's elbow and said, *“you don't have to take it if you don't want to”* [WCCAT-2], providing reassurance and respect for choice. In another observation, a nurse supporting an older person with personal hygiene asked open, relationship-building questions: *“How are you feeling today?” “Did your family have a good time overseas?”* that demonstrated attentiveness, rather than a task-orientated interaction [WCCAT-7]. Within the values and beliefs clarification activities, participants identified *“good relations”* [VBCA-2] as essential to enabling sharing decision making. Reflexive journalling by the first author during the study also illustrated the nature of relational connectedness. A creative representation showed two sets of paint colours mixing and separating, representing how values, emotions and experiences blend uniquely together in each older person and nurse relationship, reflecting both the beauty and complexity of connectedness in the context of residential aged care.

Strong relational connectedness was an enabler of meaningful participation in sharing decision making. While a weaker connection, usually resulting from rushed interactions, inauthentic presence, rigid boundaries, or organisational constraints, saw practice and interactions become more task-orientated and decisions more nurse-led. Relational connectedness was not a backdrop to sharing decision making; it was the space where knowledge of values, preferences and plans were shared and co-created. The following sections describe each of the intertwining strands influencing relational connectedness between the older person and nurse in residential aged care, and subsequently the sharing decision making process.

### 4.1 Affirming personhood

The thread of affirming personhood was described by participants as something that anchored, infiltrated and enhanced all other threads to enable sharing decision making. While participants initially referred to this component as respect, their reflections and descriptions had meaning beyond courtesy or kindness. Affirming personhood was an active, ongoing process of recognising and upholding experiences, identity and choices in everyday care interactions.

Joanne (older person) identified the importance of “*being seen as a person*” and illustrated this in her visual representation of a picture of a sky with clouds (Figure 5), representing her existing and future dreams. She explained in her interview: “*It's a nice feeling that whatever you speak up about is carried out… you’re still a person… if they put you down … you say, ‘well, what's the use?’ … and you just let yourself go*” [Joanne, older person, ETI-1]. For Joe (older person) being communicated with in Macedonian deepened his sense of identity and dignity. Affirming personhood was also evident in observed practice. During handover, nurses referred to older persons by name and described what was important to them, for example, recognising a person with dementia was experiencing distress because they were without their wallet [WCCAT-5]. Care Support Workers also described the moral weight of honouring the choices of older persons. Jess (Care Support Worker) described that when she cannot respect and act on choices: “*It doesn't make you feel very good because you’ve taken something away from what they are able to do*” [ETI-2]. Similarly, Julie (Care Support Worker) explained how important decision making and retaining control is for older people in residential aged care: “*It's everything. It's you. It's who you are … If somebody told me what I should be having, eating, doing, when I should be showered, that's everything gone for me, that's like imprisonment*” [ETI-2].

Participants also described affirming personhood as relational and reciprocal. Fred, an older person, explained: “*I think that's two-way traffic – they respect me, I respect them.*” [ETI-1]. Older persons expressed appreciation for the work of nurses [WCCAT-7], and nurses acknowledged the importance of respecting the information older persons shared with them and decisions made [Jess, Care Support Worker, ETI-2; Julie, Care Support Worker, ETI-2]. This reciprocity strengthened relational connectedness by reinforcing mutual respect. Additionally, affirming personhood was described not only as *doing* respectful actions, but also as *being* in a way that acknowledged the whole person. Elizabeth, Care Support Worker, explained: “*It's more… spiritually and emotionally connecting with the other person.*” [ETI-2].

Affirming personhood therefore was the anchor for the other threads, shaping whether trust was built, if time felt rushed or shared and whether organisational values and policies were enacted in ways that supported or undermined participation in sharing decision making. When personhood is affirmed, older persons are empowered to participate in decisions. Without affirming personhood, sharing decision making processes don't meaningfully occur, and passivity and disempowerment are felt.

### 4.2 Reciprocal trustworthiness

Participants described trust as relational and built through consistent, intentional actions by both older persons and nurses. Interestingly, trust was both expected and earned in the older person and nurse relationship. Older persons frequently spoke about trust being a condition of living in residential aged care, feeling that they had no choice but to be trusting of the nurses. Joe (older person), explained, “*they are nurses, you have to trust*” [ETI-1], while John (older person) similarly expressed, “*you come into a place like this… you have no choice but to trust them*” [ETI-1]. For others, trust was a broader reflection; Isabella (older person) described trusting others as part of her way of being: “*unless somebody does something really outrageous, I can trust them*” [ETI-2]. Fred (older person) drew on all his time living in the residential aged care setting, and said, “*I’ve been here five and a half years, and I haven't had to think otherwise*” [ETI-1], suggesting trust had been reinforced over time in his experiences of nursing care.

Nurses discussed the need to demonstrate trustworthiness through reliability and follow through. Trust was evident in many observed practices [WCCAT-1,2,3,7,8], including older persons allowing nurses to assist with personal hygiene [WCCAT-7] or taking medications dispensed by nurses without questioning [WCCAT-8]. Jess (Care Support Worker) described trust to be earned and further connected trust directly with feelings of safety: “*you’ve got to gain their trust with anything, it's really good if they have trust in you because then they will feel safe around you*” [ETI-2]. Isabella (older person) also linked her safety in her visual representation, she chose an image of a tree in a yellow field, representing the respect and trust she experienced in interactions with nurses.

Trust was described as reciprocal. Nurses expressed trust in older persons' ability to decide what was right for them, and that their role is supporting, rather than directing, choices. Julie (Care Support Worker) drew an older person in a helicopter for her visual representation (Figure 5), describing that if an older person decided they wanted to go up in a helicopter, the nurse's role was to advocate for that choice, rather than ignore or constrain it. Respecting the older person's autonomy, even where choices conflicted with safety, was seen as an important expression of trustworthiness for nurses. Trust was described to be continuously shaped and negotiated through everyday interactions; when trust was reinforced with consistency, follow through, and respectful communication, older persons engaged more openly in their care. While unreciprocated trust, or trust taken for granted, reinforces power imbalances and limits participation in sharing decision making.

### 4.3 Time as presence

Time emerged as a relational component influencing how older persons and nurses engaged in sharing decision making in residential aged care. Participants described time as an experience that held presence and attentiveness. Joanne (older person) identified “*spending the time*” was essential for building connection, describing relationships to require time to form and evolve, like the clouds Joanne chose for her visual representation (Figure 5). When nurses were authentically present and were listening, talking, or simply not rushing, older persons felt recognised and more inclined to share information relevant to decision making. Elizabeth (Care Support Worker) explained, on a good day, “*there is time… they’re listened to… they are not feeling rushed or ‘I’m just another number’*” [ETI-1]. Being present in the moment of the interaction deepened relational connectedness between the older person and nurse.

Conversely, time pressures constrained opportunities for meaningful engagement. Nurses described competing priorities and demands, staffing ratios, and routines which limited their availability and resulted in interactions shifting towards a more task focus. These time pressures were evident in observations of practice where conversation from the nurse was only about completing personal hygiene, and the older person's social conversation cues were not picked up on [WCCAT-1]. Registered Nurses also expressed frustration when information, such as vital signs or weights are needed for clinical decisions, and these aren't completed by Care Support Workers because of resourcing and workload pressures [Kim, Registered Nurse, ETI-1]. These team dynamics reduced opportunities for shared deliberation and often resulted in nurse-led or deferred decisions.

Older persons recognised nurses' competing demands on their time, and accepted delays or waiting for assistance. However, prolonged waiting influenced how respect and trust was perceived within the relationship. Isabella (older person) gave an account of waiting unclothed in the bathroom for a shower over a prolonged period, the nurse had said they would come back but then attended to someone else [ETI-2]. The nurse's prioritisation influenced her sense of importance and her comfort in future decision making encounters. The experience of time has relational consequences, shaping what and when older persons feel comfortable sharing and how willing they are to engage in sharing decision making with nurses.

Kim (Registered Nurse) identified the importance of providing older persons and their families time to reflect when making decisions: “*I gave them some space … I said, look, ‘both decisions are good decisions. Don't feel guilty if you decide to keep her here and keep her comfortable’. So, it's mainly that: giving them that time*” [ETI-1]. Enabling space and time was important for supporting autonomy and reducing guilt or pressure for decisions. Time was understood as presence instead of something being counted in minutes or hours. Brief but intentional interactions promoted relational connectedness, and rushed encounters weakened it. Time as presence therefore describes how temporal experiences influence respect, trust and participation in decision making. When time was experienced relationally, sharing decision making was strengthened.

### 4.4 Intentional way of being

Participants described that the way nurses and older persons present themselves in interactions is a deliberate choice that influences relational connectedness and engagement in sharing decision making in residential aged care. This thread in relational connectedness is about the intentional way to *be* with one another, which both older persons and nurses described as important. Nurses described choosing to project calmness, confidence, or positivity to cultivate a sense of safety and partnership. Jess (Care Support Worker) explained that she intentionally remained calm: “*If you are like angry or heightened up or anxious or anything they’ll feel the same way… so it's good just to stay calm*” [ETI-2]. Similarly, Kim (Registered Nurse) described consciously presenting as confident, even when she is feeling internally anxious, to reassure older persons and colleagues and have confidence in her clinical recommendations and decisions: “*Professionally… cool and calm and collected… I need to come across confident, even though I may not be all the time*” [ETI-1]. Joanne (older person) described “*mellowing*” her response when met with irritation by a nurse: “*If I mellow, they will*” [ETI-1]. These perspectives illustrate the relational impact of demeanour between the older person and nurse.

Observations of practice supported these descriptions of choosing an intentional way of being. During personal hygiene support, nurses were outwardly calm and unhurried despite “*busyness*” outside the room, later explaining it was intentional to give the “*impression that we aren't rushing them or thinking about other things while we wash them*” [WCCAT-3]. Georgia (Care Support Worker) linked this way of being directly to decision making, describing calmness as “*the key of communication … to find out what they want*” [ETI-2]. Participants noted that emotions spread within the practice environment. Elizabeth (Care Support Worker) explained how an older person's frustration when kept waiting could create a “*domino effect*” influencing others in their room to also get upset [ETI-1]. Intentional positive presence can be a way of shifting feelings within the communal living setting of residential aged care. Choosing to *be* calm or confident creates emotional safety and encourages communication. When demeanour conveys stress or impatience, older persons withdraw from engaging with the nurse or assumed a passive role in the relationship and sharing decision making.

### 4.5 Negotiating relational boundaries

Negotiating relational boundaries was another key component shaping relational connectedness and participation in sharing decision making. Participants described boundaries as the limits of the older person and nurse relationship, sometimes balancing the emotional with the professional. Isabella (older person) spoke about a “*fine line*” between friendliness and intrusion, explaining she was comfortable sharing her own life but would not “*dream of intruding*” into nurses' personal lives and that getting “*close, but not too close*” was, for her, “*what's healthy for you all*” [ETI-2].

For nurses, boundaries were also tied to scope of practice and role expectations. Nurses drew a clear line between *clinical* work (for example, medication management, responding to deterioration, wound care) and *care* work (such as personal hygiene support and assistance with meals) [WCCAT-2,5,7,8]. Julie (Care Support Worker) described that while respecting decisions of older person was “*paramount*”, some decisions, such as refusing medication, required her to involve a Registered Nurse (RN): “*you let the RN know what's going on and you document that*” [ETI-2]. Kim (Registered Nurse) explained that she generally interacted more deeply with older people only when a clinical issue arose [ETI-1]. This separation between *clinical* and *care* work meant that Care Support Workers often had more continuous and relational contact, while Registered Nurses had more formal and complex decision making authority.

The limits of the relationship were also negotiated when other people, such as family members or medical practitioners, were involved in decisions. Kim (Registered Nurse) described frustration when waiting for responses from general practitioners, noting how delays complicated and deferred decisions, affecting experiences and trust [ETI-1]. Isabella (older person) stated she sometimes “*thinks twice*” about raising health concerns because nurses must escalate to the doctor, adding layers to the decision making process [ETI-1]. Georgia (Care Support Worker) described it being “*awkward for us as a healthcare workers when the resident wants one way with their decision, but then the family or the next of kin wants another*” [ETI-2]. Further, Georgia explained that clear communication is important so older persons did not interpret escalation or involving others as a lack of care; she aimed to keep “*myself, resident and family members*” connected, so “*we can all make the decision with the resident together*” [ETI-2].

Cognitive function of older persons added further complexity, with some nurses equating dementia or impaired cognition with an inability to participate in any decision making process. In contrast, Julie (Care Support Worker) described expanding the relational circle to include families, “*get[ting] to know the family as much as the person*” to better support the older person living with dementia [ETI-2]. These perspectives illustrate that boundaries were not fixed, they were fluid, continually adjusted in response to role and perceived capacity. When boundaries were rigid, decisions tended to be nurse-led and hierarchical, and when they were flexible and negotiated, relational connectedness was strengthened and older persons were better able to participate meaningfully in sharing decision making.

### 4.6 Organisational values in action

Organisational values were described as significant in shaping relational connectedness between older persons and nurses, and in supporting their engagement in sharing decision making. Participants referred to organisational values as the way the mission, vision and values of the organisation influenced expectations, interactions and care culture. The residential aged care setting was part of a large faith-based organisation, and Christian values were visibly and routinely embedded in the practice environment. In observations of practice framed Bible verses and crosses displayed on the walls, and guided prayer before meetings was noted [WCCAT-3,5].

Older persons and nurses described the Christian values as enhancing a sense of belonging and acceptance, regardless of a person's own belief system. Julie (Care Support Worker) explained that the organisation's values promoted inclusivity and respect for all spiritual and religious beliefs for people of all faiths, not only those who identified as Christian [ETI-1]. Older persons reinforced this during interviews and theme-verification discussions, with Marg (older person) stating she felt valued “*no matter what*” explaining the absence of judgement and ageist attitudes was because of the organisation's values. Nurses similarly described the influence of these values, explaining that compassion and inclusion were embedded expectations, which informed how they approached interactions and decision making with older persons.

The values enacted within the practice environment influenced sharing decision making between the nurse and the older person. The emphasis on respect and inclusivity enabled an older person's preferences and beliefs to be considered in the decision making process. The promotion of a supportive and inclusive environment through the espousal and action of the Christian values made it easier for older persons to express their wishes to nurses without fear of judgement. Equally, the nurses were open to listen to the needs of the older person. The organisational values guided the interactions between nurses and older persons while meeting spiritual needs, enhancing overall wellbeing and sense of belonging within the residential aged care setting.

### 4.7 Policies in practice

Policies within the residential aged care setting shaped how older persons and nurses engaged in sharing decision making, and were referred to as the organisational rules, clinical protocols and regulatory requirements that structured everyday practice. Participants described policies as necessary for safety and consistency, but at the same time acknowledged they can hinder relational connectedness and limit autonomy. Policies about infection control were described by older persons and nurses as impacting relational connectedness. Joe (older person) described the mandated wearing of face masks during the COVID-19 pandemic by nurses made interactions difficult; English is not his primary or most comfortable language, and he relies on reading lips and facial expressions during conversations [ETI-1]. Similarly, Elizabeth (Care Support Worker) also described face masks made interactions challenging; they muffled speech, making it very difficult for older persons experiencing hearing or cognitive impairment [ETI-2]. This difficulty was also observed during a personal hygiene interaction where a nurse abandoned an attempted conversation after repeating a question three times, feeling the mask prevented meaningful engagement [WCCAT-1]. Some nurses adapted by lowering their masks momentarily or using touch and gestures to facilitate decision making; small acts of relational care that pushed against rigid policies.

Participants also identified how policies were not always meaningful in practice. In one observation, a nurse and an older person were referring to each other using “*darling*”, “*dear*” and “*sweetie*” [WCCAT-1]. In the post observation discussion, one participant initially argued that using terms of endearment with older people breached organisational policy, and others thought the older person preferred these terms, seeing it as a sign of respect [WCCAT-1]. The points raised in this discussion revealed nurses adapt policy when it doesn't feel meaningful in practice. Additionally, older persons described how policies restricted their autonomy. Isabella (older person) repeatedly referred to what she was “*allowed*” or “*not allowed*” to do, such as managing her own medications or showering independently [ETI-1,2]. She also expressed frustration at needing to ring the bell each morning to request a medication outside the scheduled round and worried she would be perceived as a complainer, which influenced how comfortable she felt voicing her needs [ETI-1]. Nurses also described navigating policies that conflicted with relational practice and flexibility in choices. Kim (Registered Nurse) explained:

…if [the advance care directive] says not for hospital, and they have said, “now I’ve changed my mind”; because we're meant to send an advance care plan with that person to hospital, I deliberately say “I don't have one”. Because otherwise [the ambulance service] end up not taking that person [to hospital], or they don't do anything for that person in hospital … I've learned, from time, to say, “don't have one, sorry” and then they have to take it as everything [the older person wanting active treatment] [Kim, Registered Nurse, ETI-1]

This situation reflected ways in which policy does not always consider changes or evolving preferences of older persons. These findings illustrate that policies in practice are negotiated rather than simply followed. Nurses and older persons continually balanced safety with autonomy and relational connectedness, often adapting or working around policies to uphold personhood and enable meaningful participation in decision making.

### 4.8 Summary

The multiple methods of information generation and collection that were synthesised using creative hermeneutic analysis, demonstrate through the findings of this research that relational connectedness is core to sharing decision making between older persons and nurses in residential aged care. The relational connectedness between older persons is shaped by multiple intertwining threads of the people, processes and structures of the practice environment (*affirming personhood, reciprocal trust, time as presence, intentional way of being, negotiating relational boundaries, organisational values in action,* and *policies in practice*). Table 2 provides a summary of each of the threads described within the findings.

**Table 2:** Summary of findings of the threads of the practice environment influencing sharing decision making between older persons and nurses.

Finding	Understanding
Relational connectedness	Sharing decision making is relational, requiring respect, reciprocal trust, knowledge sharing, and emotional engagement between older persons and nurses. Strong connections enable meaningful participation in decisions, while weaker connections lead to task-orientated or nurse-led decisions.
Affirming personhood	Respect is enacted through recognising experiences, choices and identity. When personhood is affirmed, older persons are empowered to participate in decisions; when it is not affirmed, passivity and disempowerment occur.
Reciprocal trustworthiness	Trust is built through consistent, intentional actions by older persons and nurses. Mutual trust strengthens engagement in sharing decision making, while unreciprocated trust reinforces power imbalances and weakens relational connectedness for participation in sharing decision making.
Time as presence	Time is relational rather than logistical. How time is prioritised and enacted influences relational connectedness and sharing decision making more than the quantity of time available.
Intentional way of being	The way nurses and older persons present themselves in interactions is a deliberate choice that influences relational connectedness. A positive and engaged way of being fosters trust and participation, while stress or disengagement weakens relational connectedness.
Negotiating relational boundaries	Boundaries of the relationship can be fluid, shaped by trust, autonomy and professional roles. Rigid boundaries limit engagement in sharing decision making, while flexible boundaries strengthen relational connectedness.
Organisational values in action	The values of an organisation influence relationships by shaping culture, expectations and engagement in care. When values are enacted authentically, they foster inclusivity and trust.
Policies in practice	Policies guide care but can either enable or hinder relational connectedness and sharing decision making. Nurses often adapt policies to uphold older persons’ autonomy, revealing tensions between organisational rules and realities of practice.

## 5 Discussion

**Figure 7:**
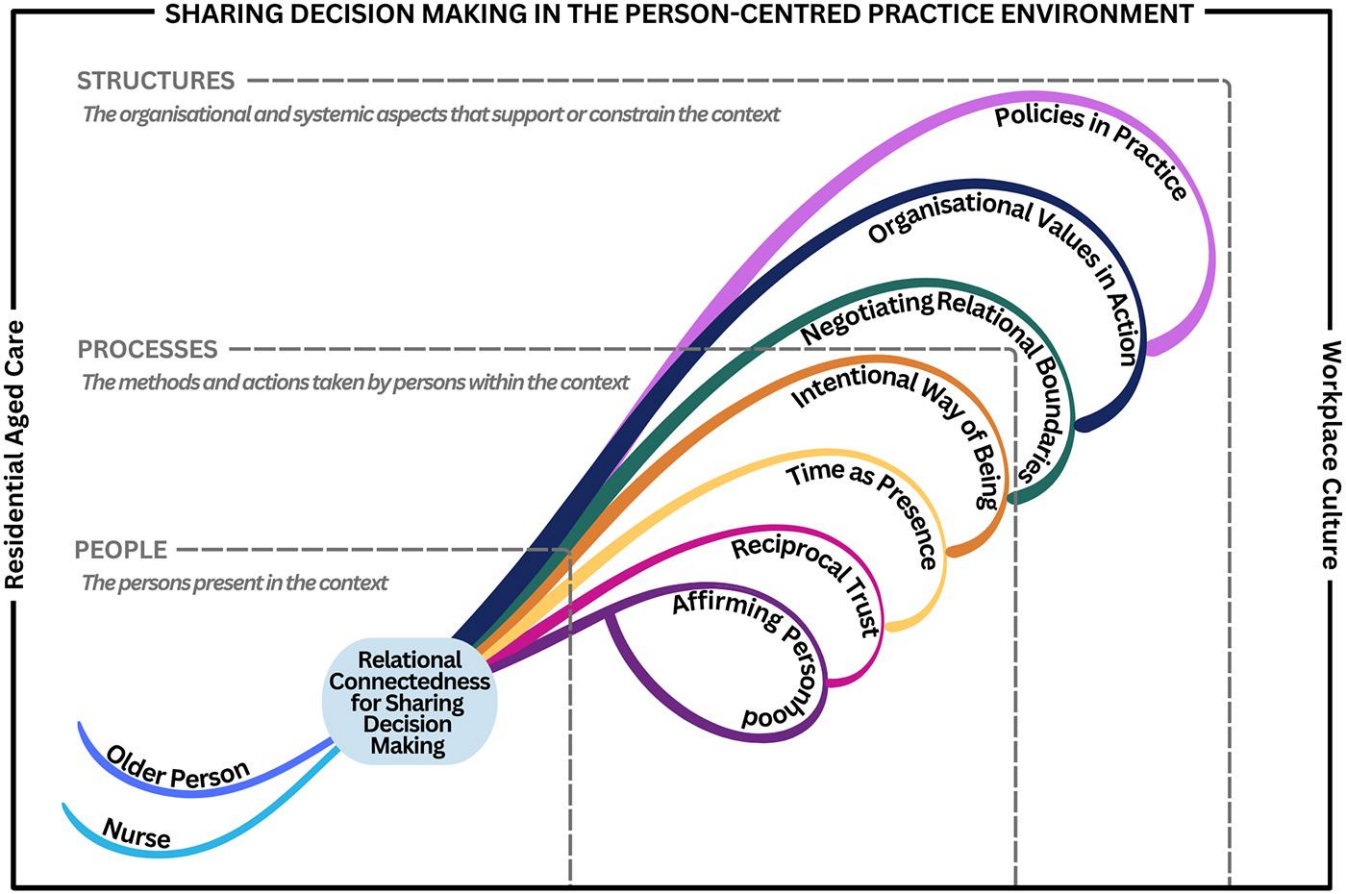
The people, processes, and structures of the practice environment influencing relational connectedness for sharing decision making.

The findings of this research illustrate that the practice environment plays a significant role in determining how, and to what extent, older persons participate in sharing decision making with nurses in residential aged care. To deepen the interpretation, the findings of this research were mapped to the constructs of the practice environment domain of the Person-centred Practice Framework to determine that sharing decision making is shaped by the people (persons present and their relationships), the processes (methods and actions taken), and the structures (organisational and systemic aspects) of the practice environment (Figure 7). In this section, the findings are discussed in relation to the people, processes and structures of the practice environment to highlight their interaction in supporting or hindering sharing decision making in residential aged care between older persons and nurses, and are summarised in Table 3.

**Table 3:** The people, processes and structures of the practice environment.

Element	Definition	Link with the practice environment domain construct of the Person-centred Practice Framework	Link with the findings (threads) of this research
People	The persons present within the context	Effective staff relationships	Relational connectedness
Processes	The methods and actions taken by the persons within the context	Power sharing	Affirming personhood
		Potential for innovation and risk taking	Reciprocal trustworthiness
			Time as presence
Structures	The organisational and systemic aspects that support or constrain the context	Appropriate skill mix	Negotiating relational boundaries
		Shared decision-making systems	Organisational values in action
		Supportive organisational systems	Policies in practice
		The physical environment	

### 5.1 The *people* within the practice environment

The *people* within the practice environment are the persons present within the context. Within this research, the *people* included older persons and nurses, as well as family members, organisational leaders, and other healthcare practitioners whose interactions influenced relational connectedness and sharing decision making. Relational connectedness emerged as central to sharing decision making. Drawing on Nussbaum's view of persons as relational, emotional beings whose flourishing depends on being in relation with others (52–54), and Fay's understanding of relationships as the point of connection between individuals and society (61); the findings reinforce that relationships in residential aged care are active, evolving, and shaped by power. The findings also support Gadow's argument that the heart of nursing lies in the intention and connection between nurse and person in care (41). Relational connectedness between older persons and nurses was strengthened by everyday acts (a calm presence, a warm greeting, following through on what was promised), and weakened by rushed or task-oriented interactions. Existing evidence identifies relational connectedness as essential to trust, respect and dignity in residential aged care (22, 81–83). This alignment may reflect the ongoing relational nature of residential aged care practice, where care is not episodic in nature. The findings extend the existing evidence by showing that relational connectedness is the condition and foundation that makes sharing decision making possible.

Nurses were key enablers of sharing decision making, but their ability to facilitate participation was dependent on the structural conditions and culture of the practice environment. Nurses enabled participation through affirming personhood, demonstrating trustworthiness, being present, and choosing an intentional way of being. While previous research has focused on the skills, knowledge, and attitudes of nurses (1, 17, 20, 84–87), these findings extend this focus by highlighting that individual capability and practice is not enough. Even the most person-centred nurse may struggle to enable sharing decision making in an environment that prioritises compliance over connection.

### 5.2 The *processes* within the practice environment

The *processes* within the practice environment refer to the methods and actions through which people interact, power is shared, and decisions unfold. Within this study, the threads of *affirming personhood, reciprocal trustworthiness, time as presence, intentional way of being* and *negotiating relational boundaries*, aligned with the constructs of *power sharing* and *potential for innovation and risk taking* within the Person-centred Practice Framework.

Affirming personhood and reciprocal trustworthiness were active processes for power sharing between older persons and nurses. Older persons described feeling they had no choice but to trust nurses when entering residential aged care, highlighting the immediate dependence and vulnerability that can accompany this transition. These findings resonate with Nussbaum's view that trust inherently involves vulnerability (88), and with Fay's argument that power is socially constructed and unequally distributed in institutional settings (61, 62). However, the findings also complicate assumptions that trust is empowering. In this study, trust only enabled sharing decision making when it was reciprocated through respectful communication, follow-through, and opportunities to express preferences, ask questions, and revisit decisions. When nurses framed choices narrowly or assumed incapacity, trust risked perpetuating passivity and power imbalance. This finding extends evidence describing paternalistic norms and constrained autonomy in residential aged care (1, 22, 36, 89), and objectifying practices of older persons (89–91): it suggests that trust and power cannot be understood separately. Trust may support participation when it is actively cultivated and shared, but it may also reinforce compliance when older persons feel dependent on nurses for everyday care. Research on the connection between trust and power in residential aged care is limited (92), and further investigation is needed to understand their influence on sharing decision making.

Time as presence further illuminated how processes shape participation. Time was experienced as relational, through how present and responsive nurses were perceived to be. When older persons were left waiting, the passage of time was interpreted as a signal that their needs, preferences, or emotions were less important, which shaped their willingness to disclose concerns or engage in decision making. This reluctance reflects research showing that older persons often adopt passive roles when they perceive nurses to be rushed or overburdened (93, 94). This similarity may be explained by the way routines and workload pressures structure time in residential aged care, which often privileges task completion over relational engagement. In contrast, even brief interactions characterised by attentiveness and follow-through reinforced relational connectedness and participation. This finding aligns with Koskenniemi et al.'s research that presence is communicated through small, intentional acts (95), and extend this work by showing that time influences decision making by how priority and presence are communicated. Time as presence therefore challenges the logistical understanding of time in residential aged care and identifies it as significant to building relational connectedness for sharing decision making.

Choosing an intentional way of being (calm, confident, mellowing) also influenced trust and openness between older persons and nurses. This perspective aligns with emotions being values-based judgments (53), and with person-centred work emphasising self-awareness and emotional intelligence as prerequisites for authentic engagement (20, 95, 96). The findings add nuance by demonstrating that older persons also chose their way of being to preserve relationships with nurses and suggests that emotional labour in residential aged care is carried by both nurses and older persons. Older persons also regulate their responses to maintain relational safety and avoid being perceived as difficult or burdensome.

Processes linked to innovation and risk taking were also evident when nurses stretched or re-interpreted policies to honour an older person's preferences. For example, choosing not to provide an advance care directive to paramedics when it no longer reflected the older person's wishes demonstrates what McCance and McCormack describe as exercising professional accountability in complex decision-making landscapes (48). This practice also reflects Baldie et al.'s view that person-centred practice often requires nurses to navigate tensions between formal frameworks and the fluid, relational nature of real-time decisions (33). These findings show that power sharing is a continuous negotiation and is either supported or constrained by organisational expectations and rigid, risk adverse cultures.

### 5.3 The *structures* within the practice environment

The *structures* refer to the organisational and systemic aspects of the context, including staffing models, policies, governance systems, and the built environment. These elements align with the Person-centred Practice Framework practice environment constructs of appropriate skill mix, shared decision-making systems, supportive organisational systems and the physical environment.

Skill mix and role delineations were among the most influential structural factors. Appropriate skill mix is defined as having the range and number of staff needed to provide quality care (48), and the findings highlight that workforce structures in residential aged care can fragment sharing decision making. Care Support Workers provided most of the day-to-day care and held strong relational connections with older persons, however, had limited decision making input or authority. Registered Nurses were responsible for a large number of older persons and held greater clinical decision making responsibility, yet had less time for relational engagement. The divide between *care* and *clinical* work was reinforced by hierarchical structures and scope of practice differences between Registered Nurses and Care Support Workers (5, 23, 28, 30), and meant older persons were not viewed in a holistic way. These findings complicate workforce discussions about staffing numbers and regulated clinical oversight. Previous research has shown that presence of Registered Nurses improves care outcomes for older persons in residential aged care (97). While Registered Nurses are fundamental for clinical safety and care quality, the findings from this study suggest that sharing decision making also depends on how relational knowledge held by Care Support Workers is recognised and incorporated into decisions. Restructuring nursing workforce skill mix and role delineation in residential aged care therefore may need to be consider more than ratios; it also requires attention to relational connectedness, decision making authority, and how different forms of nursing knowledge are valued.

Supportive organisational systems and shared decision-making systems aim to ensure safety, accountability, and collaboration (48). However, older persons in this research often experienced these systems through what they were *“allowed”* or *“not allowed”* to do. Some modifyied their requests to avoid being seen as complainers, reflecting evidence that passivity and submissiveness can become socially rewarded behaviours in institutional care (36, 89). Policies around medication administration and infection control were designed for safety and standardisation, yet sometimes undermined autonomy and connection. Additionally, nurses reported frustration when routine decisions were delayed by hierarchical approval structures or external gatekeepers, such as general practitioners. They described using workarounds when policies inhibited relational connectedness, such as momentarily removing face masks to facilitate communication. These findings reflect Fay's argument that institutional rules function as instruments of power (61), and support McCloskey's description of nurses as agents of oppression when organisational processes are applied without critical attention to their impact on older persons' autonomy (36). The findings indicate that shared decision-making systems in aged care may privilege interdisciplinary collaboration and risk management over direct collaboration with the older person, possibly limiting the very participation these systems are intended to support.

The physical environment was found to enact organisational values. In this faith-based organisation, Christian symbols, mission statements, and prayer were experienced by older persons and nurses as expressions of acceptance and belonging, which reflects evidence about the role of organisational values in shaping workplace culture (98), and research distinguishing between espoused and lived workplace values (99, 100). However, these values did not automatically neutralise structural constraints. Delays, rigid procedures, and hierarchical practices still restricted participation. This finding highlights that values must be enabled through systems and everyday relational practices if they are to support sharing decision making.

### 5.4 Implications for policy, practice, and education

The findings of this study present several opportunities to strengthen sharing decision making between older persons and nurses in residential aged care. Table 4 summarises contextually derived implications and recommendations from this research that may be transferable to similar residential aged care settings. The recommendations are informed by the findings of this study and existing evidence on person-centred practice, power sharing, and residential aged care workforce structures.

**Table 4:** Policy, education, and practice implications and recommendations for enhancing sharing decision making in residential aged care.

Area	Implications	Recommendations
Policy	Relational, procedural, and structural components of the practice environment strongly influence sharing decision making. Policies should reinforce relationality, flexibility, power sharing, and autonomy (43, 60, 61).	Embed relational connectedness into organisational policies, procedures, guidelines, and workflows. Formally recognise the relational role of Care Support Workers and enable their participation in decision making processes with older persons (5, 28, 30). Ensure organisational policies allow flexibility for older persons to revisit decisions. Review decision making structures to prevent reinforcement of hierarchical power imbalances between older persons, nurses, and other healthcare practitioners (59, 60).
Education	Emotional intelligence and understanding power dynamics are essential skills for Care Support Workers and Registered Nurses to facilitate sharing decision making (20, 94, 95).	Develop education programs for Care Support Workers and Registered Nurses focused on reflective practice and emotional intelligence that are underpinned by relational care. Embed education on power dynamics and intentional power sharing for Care Support Workers and Registered Nurses. Develop education in self-regulation, active listening, and emotional attunement to support emotionally safe spaces for decision making.
Practice	Workforce structures, culture, and the physical environment shape the relational conditions needed for shared decision processes (43, 96–99).	Ensure workforce structures (skill mix, workloads, role delineation) enable meaningful engagement in decision making with older persons (43, 96). Promote organisational cultures of relational connectedness that prioritise relationships over task-orientated practice. Design physical environments that promote communication and relational engagement that reflect values of autonomy and dignity.

### 5.5 Strengths, limitations, and recommendations for future research

The main strengths of this research are in the philosophical and theoretical congruence of the research design, which contributed to the rigour and trustworthiness of the findings (101). The research remained aligned with the ontological, epistemological, and methodological principles that guided this person-centred creative inquiry, enabling a coherent and authentic exploration of sharing decision making within residential aged care. The limitations of the study identified reflect both contextual factors and methodological constraints, which also inform future directions for research.

A perceived limitation of this research could be that information collection took place between February and July 2022, when mandated face mask wearing was in place for residential aged care settings in Australia following the COVID-19 pandemic. The requirement for face coverings made communication difficult, and as a result, may have influenced how older persons and nurses engaged in information sharing and decision making. Future research conducted outside of pandemic restrictions would provide greater clarity on how relational communication unfolds under more typical conditions. Additionally, the research was undertaken in a single residential aged care setting, and the experiences captured are specific to this context. The findings may be generalisable to similar aged care contexts, however, replicating this study across multiple locations would enable further exploration of transferability across varied settings, governance structures, and organisational cultures. Future research across diverse contexts would also support refinement and possible generalisability of the findings model.

Another limitation relates to the lack of older person and nurse involvement in the design of the research. The climate following the Royal Commission into Aged Care Quality and Safety in Australia, combined with the pressures of the COVID-19 pandemic, made it difficult to engage with residential aged care providers during the development of the research. Future studies would benefit from a co-design approach to ensure the research questions, objectives, and methods reflect the priorities and needs of those living and working in residential aged care. Recruitment of older persons through residential aged care staff may have introduced selection bias. Staff may have approached older people they perceived as more engaged or cognitively able, limiting the diversity of perspectives, particularly from older people with more complex needs or cognitive decline. Similarly, nurse recruitment may have been influenced by individual attitudes toward research, and the perspectives of more skeptical or time-pressured staff may be underrepresented. Future research could explore alternative recruitment strategies to capture a wider range of voices and reflect the complexity of residential aged care populations.

Comparative work examining how Registered Nurses and Care Support Workers uniquely influence sharing decision making would also deepen the understanding of role specific contributions in respect to their differing scopes of practice. Further research to examine how aged care governance, policy, and regulatory frameworks influence both nurse autonomy and older person participation in decisions. Studies exploring decision making capacity frameworks, particularly approaches viewing capacity as decision specific, could support a more nuanced and ethical inclusion of older persons who experience fluctuations in cognition and capacity.

Further, while this research was not explicitly designed as emancipatory, the methodology created opportunities for reflection and change among participants. Future work could explore how person-centred methodologies support transformation in relational practice, including whether reflective methodology and methods facilitate changes in perspectives or behaviours among nurses. Future research could also build on this study to develop, implement, and evaluate interventions, such as educational initiatives or organisational change strategies aimed at promoting person-centred practice and sharing decision making in residential aged care. Finally, the Person-centred Practice Framework provided a valuable structure for interpreting the findings, however, additional theoretical perspectives in future research may offer new insights to the influence of the practice environment on sharing decision making in residential aged care.

These identified limitations and recommendations are important opportunities to expand and deepen understanding of sharing decision making in residential aged care to support the development of evidence-based, person-centred practices that honour the voices and autonomy of older persons.

## 6 Conclusion

The findings of this research highlight how sharing decision making between older persons and nurses is shaped by the relational and contextual influences of residential aged care. This research demonstrates sharing decision making is a relational process, influenced by the people, processes and structures of the practice environment. The findings from this study contribute to the growing empirical evidence supporting person-centred practice and the Person-centred Practice Framework as a middle-range theory. Additionally, the findings of this research highlight the need for aged care organisations to create workforce models and structures that prioritise and enable relational connectedness between older persons and nurses. Aged care providers must review practices and policies that do not enable flexibility for decisions to be revisited or for autonomy to be enacted by older persons. Nurses should also be supported to develop reflective and power sharing practices, and emotional intelligence to facilitate participation of older persons in sharing decision making in residential aged care. The findings and recommendations from this research provide a contextually derived framework for considering the conditions that may support older persons to be respected and genuinely involved in decisions within similar residential aged care settings.

## Data Availability

The raw data supporting the conclusions of this article will be made available by the authors upon reasonable request, in accordance with ethical approvals and participant consent.

## References

[1] Ervin K Blackberry I Haines H. Shared decision making in residential aged care: a framework synthesis. Open J Nurs. (2017) 7:814–37. 10.4236/ojn.2017.77062

[2] European Network of National Human Rights Institutions. “We have the same rights”: the human rights of older persons in long-term care in Europe. Belgium. (2017). p. 1–88. Available online at: http://ennhri.org/wp-content/uploads/2019/09/Report-%E2%80%9CWe-Have-the-Same-Rights%E2%80%9D-%E2%80%93-Human-Rights-of-Older-Persons-in-Long-term-Care-in-Europe.pdf (Accessed December 15, 2025).

[3] Aged Care Quality and Safety Commission. What is person-centred care? Australian Government (2022). Available online at: https://www.agedcarequality.gov.au/resource-library/what-person-centred-care-video (Accessed December 15, 2025).

[4] Office of the High Commissioner for Human Rights. Human rights of older persons: international principles and standards - background paper. New York (2011). Available online at: https://www.ohchr.org/sites/default/files/Documents/Issues/OlderPersons/OHCHRBackgroudpaper2011.pdf (Accessed December 15, 2025).

[5] Commonwealth of Australia Royal Commission into Aged Care Quality and Safety. Final report: care, dignity and respect - volume 1: summary and recommendations. Canberra (2021). p. 1–326. Available online at: https://www.royalcommission.gov.au/aged-care/final-report (Accessed December 15, 2025).

[6] McCormack B Borg M Cardiff S Dewing J Jacobs G Janes N. Person-centredness – the ’state’ of the art. Int Pract Dev J. (2015) 5:1–15. 10.19043/ipdj.5SP.003

[7] McCormack B McCance T Martin S. What is person-centredness? In: McCormack B McCance T Bulley C Brown D McMillan A Martin S, editors. Fundamentals of Person-Centred Healthcare Practice. Hoboken: Wiley-Blackwell (2021). p. 14–22.

[8] Daly Lynn J Ryan A Kelly F. Sharing in decisions. In: McCormack B McCance T Bulley C Brown D McMillan A Martin S, editors. Fundamentals of Person-Centred healthcare Practice. Hoboken: Wiley-Blackwell (2021). p. 130–8.

[9] Elwyn G. Shared decision making: what is the work? Patient Educ Couns. (2021) 104:1591–5. 10.1016/j.pec.2020.11.032 33353840

[10] President’s Commission. President’s commission for the study of ethical problems in medicine and biomedical and behavioral research. Making health care decisions: the ethical and legal implications of informed consent in the patient–practitioner relationship. Washington (1982).

[11] Elwyn G Edwards A. Shared decision making: a path to customized rather than commercialized health care. In: Elwyn G Edwards A Thompson R, editors. Shared Decision Making in Health Care - Achieving Evidence-based patient Choice. Oxford: Oxford University Press (2016). p. 2–6.

[12] Dewing J McCormack B. Tell me, how do you define person-centredness? J Clin Nurs. (2017) 26:2509–10. 10.1111/jocn.13681 27960045

[13] McCormack B McCance T. Underpinning principles of person-centred practice. In: McCormack B McCance T, editors. Person-Centred Practice in Nursing and Health Care. Chichester: Wiley-Blackwell (2017). p. 13–35.

[14] Marriott-Statham K. The influence of the practice environment on sharing decision making between older persons and nurses in residential aged care: an Australian person-centred study (PhD thesis). Queen Margaret University, Edinburgh (2025). 10.3389/frhs.2026.1769782 PMC13323623 42395834

[15] McCormack B McCance T. Person-Centred Nursing: Theory and Practice. Chichester: Wiley-Blackwell (2010).

[16] McCance T McCormack B. The person-centred practice framework. In: McCormack B McCance T, editors. Person-Centred Practice in Nursing and Health Care. Chichester: Wiley-Blackwell (2017). p. 36–64.

[17] McCarter SP Tariman JD Spawn N Mehmeti E Bishop-Royse J Garcia I. Barriers and promoters to participation in the era of shared treatment decision-making. West J Nurs Res. (2016) 38:1282–97. 10.1177/0193945916650648 27194634

[18] Van Der Ploeg-Dorhout MP Van Den Boogaard C Reinders-Messelink H Van Der Cingel M. Patients’ experiences of shared decision-making in nursing care: a qualitative study. J Clin Nurs. (2024) 33:2274–86. 10.1111/jocn.17032 38284506

[19] Bos-van den Hoek DW Thodé M Jongerden IP Van Laarhoven HWM Smets EMA Tange D. The role of hospital nurses in shared decision-making about life-prolonging treatment: a qualitative interview study. J Adv Nurs. (2021) 77:296–307. 10.1111/jan.14549 33078865 PMC7756397

[20] Truglio-Londrigan M Slyer JT. Shared decision-making for nursing practice: an integrative review. Open Nurs J. (2018) 12:1–14. 10.2174/1874434601812010001 29456779 PMC5806202

[21] Marriott-Statham K Dickson CAW Hardiman M. Sharing decision-making between the older person and the nurse: a scoping review. Int J Older People Nurs. (2023) 18:1–12. 10.1111/opn.12507 36209506

[22] Moilanen T Kangasniemi M Papinaho O Mynttinen M Siipi H Suominen S. Older people’s perceived autonomy in residential care: an integrative review. Nurs Ethics. (2021) 28:414–34. 10.1177/0969733020948115 33000683 PMC8151558

[23] Nursing and Midwifery Board of Australia. Registered nurse standards for practice (2016). Available online at: https://www.nursingmidwiferyboard.gov.au/Codes-Guidelines-Statements/Professional-standards/registered-nurse-standards-for-practice.aspx (Accessed December 15, 2025).

[24] Nursing and Midwifery Board of Australia. Fact sheet: scope of practice and capabilities of nurses (2023). Available online at: https://www.nursingmidwiferyboard.gov.au/Codes-Guidelines-Statements/FAQ/Fact-sheet-scope-of-practice-and-capabilities-of-nurses.aspx (Accessed December 15, 2025).

[25] Australian Health Practitioner Regulation Agency. What we do (2023). Available online at: https://www.ahpra.gov.au/About-Ahpra/What-We-Do.aspx (Accessed December 15, 2025).

[26] Monro C Mackenzie L duToit S O’Loughlin K Low L-F. A preliminary exploration of the impact of aged care reforms on the governance of two Australian residential care facilities. Gerontol Geriatr Med. (2023) 9:1–10. 10.1177/23337214231176369 PMC10214066 37250600

[27] Australian College of Nursing. The role of registered nurses in residential aged care facilities: position statement (2016). Available online at: https://www.acn.edu.au/wp-content/uploads/position-statement-role-rn-residential-aged-care-facilities.pdf (Accessed December 15, 2025).

[28] Gramenz J. What is the role of an aged care worker in Australia? St Vincent’s Health Australia (2024). Available online at: https://www.svcs.org.au/people/role-of-aged-care-worker (Accessed December 15, 2025).

[29] Australian College of Care Workers. Role of personal care worker. Australian College of Care Workers. Available online at: https://www.careworkers.org.au/role-of-pcw/ (Accessed December 15, 2025).

[30] Australian College of Nursing. Regulation of the unregulated health care workforce across the health care system – a white paper. Canberra (2019).

[31] Légaré F Adekpedjou R Stacey D Turcotte S Kryworuchko J Graham ID. Interventions for increasing the use of shared decision making by healthcare professionals. Cochrane Database Syst Rev. (2018) 7:1–385. 10.1002/14651858.CD006732.pub4 PMC6513543 30025154

[32] Légaré F Thompson-Leduc P. Twelve myths about shared decision making. Patient Educ Couns. (2014) 96:281–6. 10.1016/j.pec.2014.06.014 25034637

[33] Baldie D McCance T McCormack B. Sociopolitical context in person-centred practice. In: McCormack B McCance T Bulley C Brown D McMillan A Martin S, editors. Fundamentals of Person-Centred healthcare Practice. Hoboken: Wiley-Blackwell (2021). p. 159–68.

[34] Lynch B Ryan AA O’Neill M Penney S. The factors that influence care home residents’ and families’ engagement with decision-making about their care and support: an integrative review of the literature. BMC Geriatr. (2022) 22:873. 10.1186/s12877-022-03503-8 36396991 PMC9672635

[35] McCormack B Dewing J McCance T. Developing person-centred care: addressing contextual challenges through practice development. Online J Issues Nurs. (2011) 16. 10.3912/OJIN.Vol16No02Man03 22088152

[36] McCloskey R. The ‘mindless’ relationship between nursing homes and emergency departments: what do Bourdieu and Freire have to offer? Nurs Inq. (2011) 18:154–64. 10.1111/j.1440-1800.2011.00525.x 21564396

[37] World Health Organization. Global report on ageism. Geneva (2021). Available online at: https://www.who.int/publications/i/item/9789240016866 (Accessed December 15, 2025).

[38] Kagan SH Melendez-Torres GJ. Ageism in nursing. J Nurs Manag. (2015) 23:644–50. 10.1111/jonm.12191 24238082

[39] Eliassen AH. Power relations and health care communication in older adulthood: educating recipients and providers. Gerontologist. (2016) 56:990–6. 10.1093/geront/gnv095 26491035

[40] Dong D Temple B. Oppression: a concept analysis and implications for nurses and nursing. Nurs Forum. (2011) 46:169–76. 10.1111/j.1744-6198.2011.00228.x 21806627

[41] Gadow S. Existential advocacy: philosophical foundation of nursing. In: Spicker S Gadow S, editors. Nursing Images and Ideals: Opening Dialogue With the Humanities. New York: Springer (1980). p. 79–101.

[42] Gilligan C. In a Different Voice: Psychological Theory and Women’s Development. Cambridge: Harvard University Press (1982).

[43] McCormack. Negotiating Partnerships With Older People: A Person Centred Approach. Hampshire: Ashgate Publishing Company (2001).

[44] McCormack B. A conceptual framework for person-centred practice with older people. Int J Nurs Pract. (2003) 9:202–9. 10.1046/j.1440-172X.2003.00423.x 12801252

[45] McCormack B McCance T. Person-centred Practice in Nursing and Healthcare, 2nd ed. Chichester: Wiley-Blackwell (2017).

[46] McCormack B McCance T Bulley C Brown D McMillan A Martin S. Fundamentals of Person-Centred Healthcare Practice. Hoboken: Wiley-Blackwell (2021).

[47] McCance T McCormack B Slater P McConnell D. Examining the theoretical relationship between constructs in the person-centred practice framework: a structural equation model. Int J Environ Res Public Health. (2021) 18:1–13. 10.3390/ijerph182413138 PMC8701298 34948757

[48] McCance T McCormack B. The person-centred practice framework. In: McCormack B McCance T Bulley C Brown D McMillan A Martin S, editors. Fundamentals of Person-Centred Healthcare Practice. Hoboken: Wiley-Blackwell (2021). p. 23–32.

[49] McCormack B O’Donnell D Cook N Phelan A McCance T. Strategy. In: McCormack B, editor. Developing Person-Centred Cultures in Healthcare Education and Practice: An Essential Guide. West Sussex: Wiley-Blackwell (2024). p. 29–62.

[50] Rycroft-Malone J Kitson A Harvey G McCormack B Seers K Titchen A. Ingredients for change: revisiting a conceptual framework. Qual Saf Health Care. (2002) 11:174–80. 10.1136/qhc.11.2.174 12448812 PMC1743587

[51] McCormack B Kitson A Harvey G Rycroft-Malone J Titchen A Seers K. Getting evidence into practice: the meaning of ‘context’. J Adv Nurs. (2002) 38:94–104. 10.1046/j.1365-2648.2002.02150.x 11895535

[52] Nussbaum M. Creating Capabilities: The Human Development Approach. Massachusetts: Harvard University Press (2011).

[53] Nussbaum M. Upheavals of Thought: The Intelligence of Emotions. New York: Cambridge University Press (2001).

[54] Nussbaum M. Women and Human Development. New York: Cambridge University Press (2000).

[55] Nussbaum M. Objectification. Philos Public Aff. (1995) 24:249–91. 10.1111/j.1088-4963.1995.tb00032.x

[56] Nussbaum M Levmore S. Aging Thoughtfully: Conversations About Retirement, Romance, Wrinkles, and Regret. New York: Oxford University Press (2017).

[57] de São José JMS Amado CAF. On studying ageism in long-term care: a systematic review of the literature. Int Psychogeriatr. (2017) 29:373–87. 10.1017/S1041610216001915 27852342

[58] Roberts SJ. Oppressed group behavior. Adv Nurs Sci. (1983) 5:21–30. 10.1097/00012272-198307000-00006 6410980

[59] DeMarco RF Roberts SJ. Negative behaviors in nursing. Am J Nurs. (2003) 103:113–6. 10.1097/00000446-200303000-00046 12626951

[60] Duffy E. Horizontal violence: a conundrum for nursing. Collegian. (1995) 2:5–17. 10.1016/S1322-7696(08)60093-1

[61] Fay B. Critical Social Science. New York: Cornell University Press (1987).

[62] Fay B. Contemporary Philosophy of Social Science: A Multicultural Approach. Oxford: Blackwell Publishing (1996).

[63] Jacobs G van Lieshout F Borg M Ness O. Being a person-centred researcher: principles and methods for doing research in a person-centred way. In: McCormack B van Dulmen S Eide H Skovdahl K Eide T, editors. Person-Centred Healthcare Research. Chichester: John Wiley & Sons, Ltd (2017). p. 51–60.

[64] Marriott-Statham K Mackay M Brennan N Mackay J. Empowering aged care nurses to deliver person-centred care: enabling nurses to shine. Nurse Educ Pract. (2018) 31:112–7. 10.1016/j.nepr.2018.05.014 29857277

[65] Dewing J. Participatory research: a method for process consent with persons who have dementia. Dementia. (2007) 6:11–25. 10.1177/1471301207075625

[66] Dewing J. Process consent and research with older persons living with dementia. Res Ethics. (2008) 4:59–64. 10.1177/174701610800400205

[67] Sieber J Tolich M. Communicating informed consent and process consent. In: Sieber J Tolich M, editors. Planning Ethically Responsible Research. California: SAGE Publications (2013). p. 115–40.

[68] Dewing J McCormack B Titchen A. Practice Development Workbook for Nursing, Health and Social Care Teams. Oxford: Wiley-Blackwell (2014). 10.7748/nop.26.8.11.s15 25258230

[69] Creswell J Poth C. Designing a qualitative study. In: Creswell J Poth C, editors. Qualitative Inquiry and Research Design, 4th ed. California: SAGE Publications (2018). p. 41–63.

[70] Schubotz D. Research ethics in participatory research practice. In: Schubotz D, editor. Participatory Research: Why and How to Involve People in Research. London: SAGE Publications (2020). p. 69–94.

[71] Dewar B Nolan M. Caring about caring: developing a model to implement compassionate relationship centred care in an older people care setting. Int J Nurs Stud. (2013) 50:1247–58. 10.1016/j.ijnurstu.2013.01.008 23427893

[72] Dewar B Mackay R Smith S Pullin S Tocher R. Use of emotional touchpoints as a method of tapping into the experience of receiving compassionate care in a hospital setting. J Res Nurs. (2010) 15:29–41. 10.1177/1744987109352932

[73] Health Improvement Scotland. Emotional touchpoints (2025). Available online at: https://www.hisengage.scot/engaging-communities/participation-toolkit/emotional-touchpoints/ (Accessed December 15, 2025).

[74] Wilson V Dewing J Cardiff S Mekki TE Øye C McCance T. A person-centred observational tool: devising the workplace culture critical analysis tool®. Int Pract Dev J. (2020) 10:1–15. 10.19043/ipdj.101.003

[75] McCormack B Henderson E Wilson V Wright J. Making practice visible: the workplace culture critical analysis tool (WCCAT). Pract Dev Health Care. (2009) 8:28–43. 10.1002/pdh.273

[76] Dickson C van Lieshout F Kmetec S McCormack B Skovdahl K Phelan A. Developing philosophical and pedagogical principles for a pan-European person-centred curriculum framework. Int Pract Dev J. (2020) 10:1–20. 10.19043/ipdj.10Suppl2.004

[77] Boomer C McCormack B. Creating the conditions for growth: a collaborative practice development programme for clinical nurse leaders. J Nurs Manag. (2010) 18:633–44. 10.1111/j.1365-2834.2010.01143.x 20840357

[78] Sanders K Marriott-Statham K Mackay M McMillan A Rennie K Robinson BA. The student international community of practice: a critical reflection on the shared experience of being a member, using creative hermeneutics. Int Pract Dev J. (2020) 10:1–10. 10.19043/ipdj.101.011

[79] Bartley KA Brooks JJ. Fusion of horizons: realizing a meaningful understanding in qualitative research. Qual Res. (2021) 23:1–22. 10.1177/14687941211065164

[80] Gadamer H. Truth and Method. London: Sheed and Ward (1993).

[81] Moore K Kelly F. Experiencing person-centredness in long-term care. In: McCormack B McCance T Bulley C Brown D McMillan A Martin S, editors. Fundamentals of Person-Centred Healthcare Practice. Hoboken: Wiley-Blackwell (2021). p. 199–208.

[82] Younas A Porr C Maddigan J Moore J Navarro P Whitehead D. Behavioural indicators of compassionate nursing care of individuals with complex needs: a naturalistic inquiry. J Clin Nurs. (2023) 32:4024–36. 10.1111/jocn.16542 36123303

[83] McCormack B Roberts T Meyer J Morgan D Boscart V. Appreciating the ‘person’ in long-term care. Int J Older People Nurs. (2012) 7:284–94. 10.1111/j.1748-3743.2012.00342.x 23164250

[84] Ervin K Blackberry I Haines H. Developing a taxonomy and mapping concepts of shared decision making to improve clinicians understanding. Nurs Care Open Access J. (2017) 3:1–7. 10.15406/ncoaj.2017.03.00063

[85] Cranley LA Slaughter SE Caspar S Heisey M Huang M Killackey T. Strategies to facilitate shared decision-making in long-term care. Int J Older People Nurs. (2020) 15:1–10. 10.1111/opn.12314 PMC7507187 32196984

[86] Clark NM Nelson BW Valerio MA Gong ZM Taylor-Fishwick JC Fletcher M. Consideration of shared decision making in nursing: a review of clinicians’ perceptions and interventions. Open Nurs J. (2009) 3:65–75. 10.2174/1874434600903010065 19855848 PMC2765030

[87] Stirling C. Communication and shared decision-making. In: Vafeas C Slatyer S, editors. Gerontological Nursing: A Holistic Approach to the Care of Older People. Chatswood: Elsevier (2021). p. 63–70.

[88] Nussbaum M. Anger and Forgiveness: Resentment, Generosity, Judgement. New York: Oxford University Press (2016).

[89] Molina-Mula J Gallo-Estrada J. Impact of nurse-patient relationship on quality of care and patient autonomy in decision-making. Int J Environ Res Public Health. (2020) 17:1–24. 10.3390/ijerph17030835 PMC7036952 32013108

[90] Band-Winterstein T. Health care provision for older persons: the interplay between ageism and elder neglect. J Appl Gerontol. (2015) 34:113–27. 10.1177/0733464812475308 24652870

[91] Wikström E Emilsson UM. Autonomy and control in everyday life in care of older people in nursing homes. J Hous Elderly. (2014) 28:41–62. 10.1080/02763893.2013.858092

[92] Möllering G. Connecting trust and power. J Trust Res. (2019) 9:1–5. 10.1080/21515581.2019.1609732

[93] Oxelmark L Ulin K Chaboyer W Bucknall T Ringdal M. Registered nurses’ experiences of patient participation in hospital care: supporting and hindering factors patient participation in care. Scand J Caring Sci. (2018) 32:612–21. 10.1111/scs.12486 28675925

[94] Penney W Wellard SJ. Hearing what older consumers say about participation in their care. Int J Nurs Pract. (2007) 13:61–8. 10.1111/j.1440-172X.2006.00608.x 17244246

[95] Koskenniemi J Leino-Kilpi H Suhonen R. Manifestation of respect in the care of older patients in long-term care settings. Scand J Caring Sci. (2015) 29:288–96. 10.1111/scs.12162 25213177

[96] Lynch B Barron D McKinlay L. Connecting with others. In: McCormack B McCance T Bulley C Brown D McMillan A Martin S, editors. Fundamentals of Person-Centred Healthcare Practice. Hoboken: Wiley-Blackwell (2021). p. 93–101.

[97] Spilsbury K Charlwood A Thompson C Haunch K Valizade D Devi R. Relationship between staff and quality of care in care homes: staRQ mixed methods study. Health Soc Care Deliv Res. (2024) 12:1–139. 10.3310/GWTT8143 38634535

[98] Seah SSL Chenoweth L Brodaty H. Person-centred Australian residential aged care services: how well do actions match the claims? Ageing Soc. (2022) 42:2914–39. 10.1017/S0144686X21000374

[99] Manley K Saunders K Cardiff S Webster J. Effective workplace culture: the attributes, enabling factors and consequences of a new concept. Int Pract Dev J. (2011) 1:1–29. https://www.fons.org/wp-content/uploads/2024/03/IPDJ_0102_01

[100] Cardiff S Sanders K Webster J Manley K. Guiding lights for effective workplace cultures that are also good places to work. Int Pract Dev J. (2020) 10:1–20. 10.19043/ipdj.102.002v2

[101] Guba E Lincoln Y. Fourth Generation Evaluation. Thousand Oaks: Sage Publications (1989).

